# Iron Status, Anemia, and Iron Interventions and Their Associations with Cognitive and Academic Performance in Adolescents: A Systematic Review

**DOI:** 10.3390/nu14010224

**Published:** 2022-01-05

**Authors:** Kaitlyn L. I. Samson, Jordie A. J. Fischer, Marion L. Roche

**Affiliations:** 1 Food, Nutrition, and Health, University of British Columbia, Vancouver, BC V6T 1Z4, Canada; kaitlyn.samson@ubc.ca (K.L.I.S.); jordie.fischer@ubc.ca (J.A.J.F.); 2 Healthy Starts, British Columbia Children’s Hospital Research Institute, Vancouver, BC V5Z 4H4, Canada; 3 Nutrition International, Ottawa, ON K2P 2K3, Canada

**Keywords:** iron, anemia, cognitive performance, academic performance, adolescents, education

## Abstract

In adolescents, iron-deficiency anemia is the leading cause of disability-adjusted life years lost. The World Health Organization recommends delivering iron supplementation through school-based platforms, requiring partnerships with the education sector. This anemia-reduction intervention is valued for the perceived benefits of improved learning and school performance. This article aims to systematically review the available evidence on the relationship between iron status and anemia and impacts of iron interventions on cognitive and academic performance in adolescents. Fifty studies were included: *n* = 26 cross-sectional and *n* = 24 iron-containing interventions. Our review suggests that iron status and anemia may be associated with academic performance in some contexts and that iron supplementation during adolescence may improve school performance, attention, and concentration. However, nearly all supplementation trials were judged to have moderate or high risk of bias. We did not find evidence suggesting that iron status and anemia influenced or were associated with attention, intelligence, nor memory in adolescents. Further, iron supplementation did not improve memory and recall or intelligence. Overall, more high-quality research is needed to guide programmers and policy makers to understand the relationships between anemia and educational performance and the potential impacts of iron interventions, which effectively reduce anemia, on adolescents’ learning and school performance.

## 1. Introduction

Iron is an essential trace mineral required for a variety of functions; it plays a role in not only brain development but also brain function [1]. Iron deficiency (ID) is commonly due to inadequate dietary intake of bioavailable iron; however, low iron status can also happen during periods of increased growth requirements, such as early in childhood and adolescence when red blood cell mass is expanding [1,2]. Anemia, the most marked consequence of ID, is characterized by a reduction in the blood’s oxygen-carrying capacity. This can lead to symptoms such as fatigue, weakness, and reduced work capacity [3]. In some instances, behavioral disturbances and impaired performance in cognitive tasks can occur [2]. This, in turn, may lead to poor academic performance. In adolescents, iron-deficiency anemia (IDA) is estimated to be the leading cause of disability-adjusted life years (DALYs) lost in boys and girls aged 10–14 years and girls aged 15–19 years [4].

Prior reviews have found that academic achievement is positively associated with breakfast consumption and global diet quality/meal patterns and negatively associated with junk/fast food consumption [5]. In a review of older children and adults, it was found that iron supplementation improved attention and concentration regardless of baseline iron status and that, in anemic participants, intelligence quotient (IQ) improved by 2.5 points (95% CI: 1.24, 3.76) [6]. Reviews of anemic infants and children have found that iron supplementation positively affects cognition and psychomotor outcomes [7], including global cognitive score, IQ, and attention and concentration [8]. Overall, these results suggest that targeting iron supplementation programs to adolescents may improve academic performance, in addition to improving iron status. However, adolescents are an often-overlooked group for which data are lacking to inform effective policy. Thus, this review aims to determine current evidence on (1) the relationship between anemia and iron status and adolescents’ cognitive and academic performance and (2) the effect of iron interventions, specifically supplementation and fortification, on adolescents’ cognitive and academic performance.

## 2. Materials and Methods

This systematic literature review is reported according to the Preferred Reporting Items for Systematic Reviews and Meta-Analyses (PRISMA) guidelines. The review protocol is registered with PROSPERO (CRD42020158754).

### 2.1. Search Strategy

MEDLINE, EMBASE, and CINAHL were searched for studies to include in this systematic review up to and including 5 December 2019 (Supplementary Tables S1–S3). Google Scholar, Web of Science, and Cab direct were used to search the Grey literature. The search strategy was made in consultation with a librarian. Cited reference searches and reference lists of included studies were examined for any other relevant articles. Articles were restricted to those on humans, and no restrictions for publication dates or language were used. Due to conflicting search engine definitions of the adolescent period, no restrictions for age were used, and all individual articles were inspected for eligible age ranges.

### 2.2. Selection Criteria and Eligibility

Primary research articles, consisting of randomized controlled trials, cohort studies, and cross-sectional studies, were included in this review. Eligible studies had to include adolescents; people 10–19 years of age as defined by the World Health Organization (WHO). Articles that did not specifically recruit from this age range but had overlapping age ranges were also examined, and those with disaggregated adolescent data were eligible. Papers that had a mean age that fell between 10 and 19 years were also eligible for inclusion. Articles were eligible if they included a biochemical marker of iron or anemia status, such as ferritin, soluble transferrin receptor (sTfR), serum iron (SI), total iron-binding capacity (TIBC), transferrin saturation (TS), and/or hemoglobin (Hb) concentration; an estimated intake of dietary iron; or a supplementation or fortification intervention that included iron. Eligible outcomes included cognitive function, learning, academic performance, memory, thinking, attention, and concentration.

Two reviewers (KLIS and JAJF) were responsible for independently screening and reviewing the title and abstracts of the search results. Full-text articles were obtained from relevant citations to determine eligibility. In instances where the full-text copies could not be accessed, authors were contacted to obtain a full text. If there was no response, “unable to obtain full text” was noted as an exclusion reason. Disagreements about study eligibility were resolved through discussion and consultation with a third party (MLR).

### 2.3. Quality Assessment

Two reviewers (KLIS and JAJF) were responsible for assessing the articles, as guided by the PRISMA checklist (Supplementary Table S4). Quality assessment and risk of bias were conducted using the revised Cochrane Risk of Bias tool (RoB 2.0) for randomized trials [9]. The Cochrane Risk of Bias system includes five domains that are evaluated as having a low, high, or unclear risk of bias. The Risk of Bias In Non-randomized Studies of Interventions (ROBINS-1) tool was used for included non-randomized intervention studies [10]. For non-randomized observational studies, the National Heart, Lung and Blood Institute’s (NHLBI) Quality Assessment Tool for Observational Cohort and Cross-Sectional Studies was used to assess methodological quality [11].

### 2.4. Data Extraction and Synthesis

Information from eligible studies was recorded into evidence tables. Information summarized included the study design, setting, year, country, population, sample size, exposure, duration of exposure, exposure assessment method, outcome assessment method, and measures of association. Data were extracted by one reviewer and verified by a second reviewer. A synthesis of the existing evidence was conducted; however, due to extensive heterogeneity in outcome measures, a meta-analysis was not possible with the available data for this systematic review.

## 3. Results

**Figure 1:**
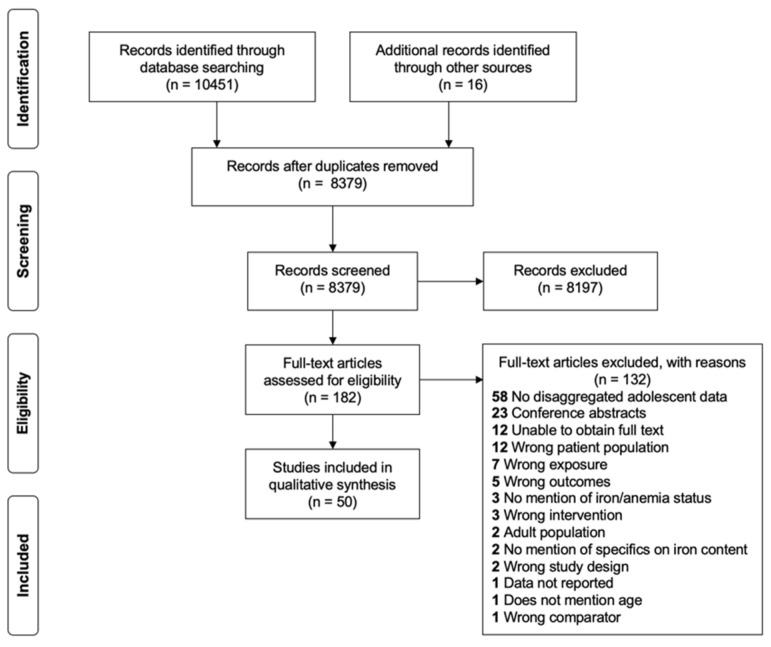
PRISMA flow diagram of study selection.

There were 10,451 articles and abstracts initially retrieved from the database search, and 16 articles were identified from additional sources. After removing duplicates, 8379 titles and abstracts were screened for eligibility. One hundred and eighty two articles underwent full-text screening, and 132 papers were excluded based on the aforementioned eligibility criteria. Fifty articles were selected for inclusion in this systematic review (Figure 1).

### 3.1. Description of Included Studies

In total, there were 26 eligible cross-sectional studies (Table 1) and 24 eligible iron-containing intervention studies (Table 2). Four of the intervention studies were cluster randomized [12,13,14,15], and one study was a non-randomized placebo-controlled study [16]. One of the intervention trials did not elucidate a significant change in iron status [14]; thus, the researchers did not investigate the associations between concomitant changes during the study. Only baseline associations between cognition and iron status were assessed for this study. One of the cross-sectional studies included was the baseline analyses from a cluster-randomized control trial [17]. The full trial was not included in this review as it included an intervention outside the scope of this review.

Experimental studies included a wide variety of interventions that aimed to increase iron intake. However, the common themes included iron supplementation; food-based interventions that consisted of food fortified with iron in some capacity; promotion or provision of iron-rich foods with known iron content; and iron supplements that also contained additional micronutrients. Iron-only supplementation interventions had various doses, ranging from 2 mg of elemental iron to 260 mg of elemental iron daily. Ferrous sulphate was the most common form of iron used (5 out of 9 studies).

Multiple micronutrient supplements contained anywhere from 2.5 mg of iron to 36 mg of iron daily, along with at least one and up to 22 additional vitamins and minerals. Eight interventions were food-based. Three interventions provided micronutrient-fortified powdered drinks with iron contents ranging from 7.0 mg given six days per week to 14 mg of iron given twice daily [15,44,47]. Two interventions provided fortified and biofortified wheat (6 mg iron) and pearl millet (86.3 ppm iron), respectively [45,46]. One study provided school meals cooked with multiple micronutrient fortified salt (10 mg of elemental iron daily per person) [62]. One study provided a nutritional ball made of roasted rice flakes and jaggery (non-centrifugal cane sugar, 13.14 mg iron) [43], and one study provided 1–2 tablespoons of *Ragi* powder (finger millet) twice per day (an estimated 0.58–2.12 mg iron per day) [12].

Participants of the included studies ranged in age from 5 to 22 years, but all included studies had a mean age between 10 and 19 years. Sixty-six percent of the studies (*n* = 33) included both male and female participants, while the remaining 34% (*n* = 17) included only female participants. Thirty-four percent of the studies (*n* = 17) were conducted in high-income countries (Chile, Denmark, England, Israel, Italy, New Zealand, Saudi Arabia, South Korea, Spain, and the USA) [63]. Eighteen percent (*n* = 9) of the studies took place in upper-middle-income countries (China, Iran, Jamaica, Malaysia, and Venezuela) [63]. Forty-six percent (*n* = 23) were conducted in low-middle-income countries (Bangladesh, India, Indonesia, Kenya, Morocco, Sri Lanka, and the Philippines), and one study was conducted in a low-income country (Ethiopia) [63]. Based upon the outcomes of interest, data from 26,291 participants were included in this review. Of these participants, 13,641 were from high-income countries, 4266 were from upper-middle-income countries 7942 were from low-middle-income countries, and 442 were from the low-income country. Two of the included articles were translated from Spanish by one reviewer (KLIS) [22,36].

### 3.2. Quality Assessment and Risk of Bias

The Cochrane RoB 2.0 summary of the included randomized intervention trials is detailed in Table 3 [9]. The studies were judged to be at varying levels of risk of bias, with nine having a high risk of bias, seven having an unclear risk of bias, and seven with a low risk of bias. The studies with an unclear risk of bias commonly lacked information regarding participants; had unblinded delivery of the intervention; or did not analyze the data as per a pre-specified analysis plan. The risk of bias summary for the one non-randomized study is detailed in Table 4 [10]. The study was judged unclear as there was a lack of adjustment for potential confounding variables [16]. The study quality assessment summary for the included cross-sectional studies is found in Table 5 [11]. Most studies were judged to have an overall poor-quality rating due to various reasons, such as unclear information regarding the participation rate and the total population size screened from, lack of sample size justification, no information regarding the blinding process, and not adjusting for confounders in the analysis. Further, because of the cross-sectional design of the included studies, nearly all studies received “no” ratings for the exposure being assessed before the outcome measure, sufficient timeframe to see an effect, and repeated exposure assessments conducted.

### 3.3. Association of Iron Status and/or Anemia with Dimensions of Academic Performance and Cognitive Function 

Overall, 25 studies examined the relationship between iron status and/or anemia and measures of cognitive performance or school achievement [14,17,18,19,21,22,23,24,25,26,27,29,30,31,32,33,34,35,36,37,38,39,40,41,42].

#### 3.3.1. Attention and Concentration

Five studies assessed attention and concentration [14,17,32,36,38]. In two studies, anemia and iron status were found to be associated with reduced attention and concentration performance [14,32]. One study found lower attention scores among males with ID; however, this did not extend to female participants [36]. Two studies found no association between iron status and anemia with attention and concentration [17,38].

#### 3.3.2. Intelligence

In total, 13 studies reported on measures of intelligence. Five studies found differences in measures of intelligence based on iron and anemia status [19,24,32,35,40]. Three of these studies assessed intelligence using the Raven’s Progressive Matrices (RPM), a widely used non-verbal group test of fluid intelligence, and found that anemic children performed worse than non-anemic children [19,24,40]; however, it should be noted that Anuar Zaini et al. did not conduct any statistical analyses. Using the Bhatia battery performance test, consisting of Kohs’ block design (for visuo-constructional ability) and the Passalong test (for concrete ability), More et al. found that IQ scores were lower in both anemic and non-anemic iron-deficient girls, as compared with non-iron-deficient girls [32]. Olson et al. used the Philippines non-verbal intelligence test and found that students with IDA and anemia had significantly lower non-verbal intelligence scores than students with no anemia [35].

Three studies showed mixed results for the effects of ID on intelligence [26,36,37]. Two of these studies used the Wechsler Intelligence Scale for Children (WISC), one of the most commonly used youth intelligence tests [26,37]. Halterman et al. found that children with IDA or ID did not have significantly different scores than children with normal iron status, on both reading and digit span tests. For reading, block design, and digit span (a working memory subtest), the percentage of children scoring below average did not differ by iron status. Children with ID were not at increased odds of scoring below average for reading, block design, or digit span (adjusted analyses, *p* > 0.05). However, children with IDA scored lower than children with normal status for block design (8.0 vs. 9.5, *p* < 0.05) [26].

Sen et al. reported that girls without anemia performed significantly better on the digit span test and visual memory tests in both the 9–11 age range and the 12–14 age range. However, performances on the maze test (for visual–motor coordination) and clerical task (for concentration and ability to discriminate) did not differ by anemia status [37]. Using an adaptation of the SRA Test of Educational Ability, Ortega et al. found that, in girls, the presence of ID was associated with significantly lower IQ scores and that there was a significant difference in Hb levels among IQ percentiles. However, there was no significant difference in IQ scores between iron-deficient and non-iron-deficient boys. Ferritin levels were significantly positively associated with IQ percentile among boys [36].

Four studies reported no difference in IQ between varying statuses of anemia and ID [21,23,25,29]. Halliday et al. reported that the presence of anemia was not associated with a difference in literacy performance, non-verbal reasoning, comprehension, or numeracy skills [17].

#### 3.3.3. Memory and Recall

Four studies examined memory and recall [32,35,38,40]. Two studies found no correlation with memory [38,40]. One study had mixed results, finding that children with anemia scored worse than non-anemic children for verbal memory on the Wide-Range Assessment of Memory and Learning (WRAML) Verbal Memory Index after adjusting for confounding variables. On the same test, children with IDA scored 2.8 corrected points lower than non-anemic children, but this was not significant (*p* = 0.12) [35]. Additionally, anemia, regardless of type, had no significant effect on verbal fluency, a measure of working memory, after adjustment [35]. Only one study reported that verbal memory and recognition were lower in iron-deficient girls, both anemic and non-anemic, as compared with the non-iron-deficient girls [32].

#### 3.3.4. School Performance

Sixteen studies reported measures of school performance. Four studies found a negative effect of anemia on school test scores and achievement [18,34,39,41]. Anemic students made up a greater proportion of those with “fail/pass” grades, compared with those with “excellent grades” (no statistical analyses reported) [18]. Additionally, anemia was associated with lower achievement levels in reading and spelling [41]. One study found that anemia and IDA negatively impact school test scores, but this did not extend to the subjects with ID without anemia [34]. Two studies reported lower scholastic performance in iron-deficient girls, both anemic and non-anemic, compared with the non-iron-deficient girls [26,32].

Two studies reported significant positive associations between Hb concentrations and aspects of school achievement [24,27]. Hutchinson et al. found that Hb had significant positive correlations with reading and spelling scores; however, it was not correlated to math scores [27]. Alternatively, El Hioui et al. found that math GPA was significantly related to Hb level (*p* = 0.048). Furthermore, ferritin was positively correlated with GPA in math (*r* = 0.5, *p* < 0.05) and cumulative GPA (*r* = 0.37, *p* < 0.05) [24]. However, it should be noted that the model was not adjusted for other covariates.

Two studies reported results that differed by sex [14,36]. The first study reported that in girls, ID was associated with significantly lower school aptitude scores; however, there was no association with iron status and individual subject grades [36]. In boys, there was a significant association between ID and lower school aptitude scores as well as lower grades in physics and chemistry [36].

The second study found that low iron stores (defined here as ferritin ≤ 25 µg/L due to the low number of children with iron deficiency as ferritin < 15 µg/L) were associated with worse school performance in girls but not boys (*p* = 0.033) [14]. On the sentence reading test, girls with low iron stores had lower reading speed and number of correct sentences compared with iron-replete girls. In contrast, boys with low iron stores had higher reading speed and number of correct sentences than iron-replete boys. Small iron stores were also associated with improvement in reading accuracy for both sexes. Additionally, an association was found between baseline ferritin status and reading comprehension (*p* < 0.035). This association indicated better performance in children with small iron stores compared with iron sufficient children, and the association was not different between sexes. Iron stores were not associated with performance on a math test (*p* = 0.141), which was also not significant between sexes (*p* = 0.93) [14].

One study reported that anemic subjects had better school achievement [42]. Webb et al. found that 12-year-old anemic girls scored better than non-anemic girls for their composite score on the Iowa Tests of Basic Skills, a statewide measure of scholastic performance that consisted of vocabulary, reading comprehension, spelling and grammar, map-graph table reading, knowledge and use of reference materials, arithmetic concepts, and problem-solving subtests. Overall, anemic subjects differed from non-anemic subjects in composite score achievements (*p* < 0.025). In all other instances, both male and female anemic subjects scored worse than non-anemic subjects [42].

Three studies found no influence of iron or anemia status on school performance [21,22,23]. One study reported that of the 14 children with anemia, six had low academic achievement scores (42.9%), although no statistical analyses were reported [31]. Anuar Zaini et al. found that students with severe anemia performed better than those with normal Hb status for Malay language comprehension, Malay language written, math, and English. However, no statistical analyses to demonstrate significant differences were reported either [19].

#### 3.3.5. Other Measures of Cognitive Performance

Four studies reported on additional measures of cognitive performance [29,30,32,33]. Two studies reported on reaction times [29,33]. One showed both low and high SI resulted in slower test speeds and reaction time compared with students with normal SI status. The students with high SI also had decreased abstraction ability and mental flexibility [29]. Another found a negative correlation between Hb and whole-body reaction time (*p* = 0.000) [33]. One study reported scores of mental balance, which were decreased in iron-deficient girls, both anemic and non-anemic, as compared with the non-iron-deficient girls [32]. The final study examined P300 (a measure of stimulus evaluation or categorization) latency and amplitude. The latency of P300 was found to be significantly delayed in the anemic group and amplitude was significantly higher in the control group, demonstrating worse performance in the anemic group [30].

### 3.4. The Effects of Dietary Iron Intake on Dimensions of Academic Performance and Cognitive Function

Three studies reported the effects of dietary iron intake on school achievement and cognitive performance [20,28,36].

#### 3.4.1. School Performance

Two studies assessed dietary iron intake and school performance [20,28]. One study examined estimated daily iron intake in mg, which was higher among those with satisfactory school performance; here, iron intake was positively correlated with written math, oral math, and written Italian scores [20]. The remaining study assessed iron intake as the % daily value, which was significantly correlated with scholastic achievement, yet no confounders were adjusted for in this model [28].

#### 3.4.2. Intelligence

One study reported on the effects of self-reported iron intake using the 5 day “food consumption registration” technique on intelligence, with no associations observed between iron intake (mg/day) and IQ percentile [36].

### 3.5. The Effects of Food-Based Interventions on Dimensions of Academic Performance and Cognitive Function 

Eight studies examined the effects of food-based and fortification-based interventions on school performance and cognitive function [12,15,43,44,45,46,47,48].

#### 3.5.1. Attention and Concentration

Three studies reported the effects of food-based interventions on attention and concentration [15,44,46]. One study found that the intervention led to improvements in attention and/or concentration [46]. The second study reported mixed results, with the intervention group having superior performance on the Knox Cube Imitation Test, requiring the ability to perceive and remember sequences of increasing difficulty. In contrast, the placebo group performed better on the Letter Cancellation Test, where only recent memory and recall of the specified letters were required [15]. The third study found a negative intervention effect on sustained attention. The placebo group performed significantly better than the intervention group on the visual search test, which measures visual information processing speed and sustained attention [44].

#### 3.5.2. Intelligence

Five studies assessed the effects of food-based interventions on intelligence [15,43,44,45,47]. Two studies used the Malin’s Intelligence Scale for Indian Children, an adaptation of the WISC [15,43]; one study measured intelligence using Raven’s Colored Progressive Matrices (RCPM) [44]; one study used the Primary Mental Abilities Test for Filipino Children, a standardized written test for verbal, non-verbal, and cognitive ability [47]; and one study selected seven different cognitive tests to obtain an overall cognitive score [45]. Kalaichelvi concluded that there was a positive effect of the intervention on intelligence [43], while three of the other studies saw no effect on markers of intelligence [15,44,45]. The remaining study found no significant effect of treatment on overall scores; however, non-verbal ability scores significantly improved among intervention subjects who were moderate to severely anemic at baseline [47].

#### 3.5.3. Memory and Recall

Four studies investigated the effects of food-based interventions on memory [15,44,46,48]. Two of these studies revealed a positive effect of food-based interventions on memory [46,48], while the remaining two studies saw no improvements in memory following intervention [15,44].

#### 3.5.4. School Performance

Three studies analyzed the effects of food-based interventions on school performance [12,15,44]. One study examined the effects of *Ragi* powder [12]; another assessed the effects of an orange-flavored micronutrient-fortified powdered beverage mix [44]; and the third examined a health drink with a micronutrient supplement (the drink formulation contained wheat flour, malted barley, skimmed milk powder, and sugar) [15]. Two of the studies found no significant changes in school performance following the interventions [12,15]. At the same time, the third study observed a higher achievement in spelling and math among the intervention group as compared with the control group [44].

#### 3.5.5. Other Measures of Cognitive Performance

One study measured reaction time; here, it was found that reaction time decreased twice as much from 0 to 6 months in those consuming biofortified pearl millet compared with those consuming conventional pearl millet on attention tasks [46].

### 3.6. The Effects of Iron-Only Supplementation Interventions on Dimensions of Academic Performance and Cognitive Function 

Overall, nine studies reported the effects of iron-only supplementation interventions on school achievement and cognitive performance [16,49,50,51,52,53,54,55,56]. Intervention duration ranged from 8 weeks to 8 months, and iron doses ranged from 2 mg of elemental iron per day to 260 mg elemental iron per day. Two interventions did not follow a daily regimen; one study provided 100 mg elemental iron six days per week, and the other provided 50 mg iron two days per week [16,53].

#### 3.6.1. Attention and Concentration

Four studies examined the effects of iron supplementation on attention and concentration. Two of the studies found a significant positive effect of iron supplementation on concentration [53,55] and one study found an improvement in the reported ability to concentrate in school (self-reported) following the iron intervention [49]. The final study found that iron supplementation did not affect attention test scores [50].

#### 3.6.2. Intelligence

Three studies reported on the effects of iron supplementation on intelligence [16,54,56]. Two of these studies used the RPM to measure intelligence [16,54]. Overall, two studies reported improvements in IQ compared with the control groups [16,56], and one study reported that supplementation had no significant effect on IQ [54].

#### 3.6.3. Memory and Recall

Five studies assessed the effects of iron supplementation on memory and recall [16,50,51,52,56]. A positive effect of iron supplementation on memory was reported in three studies [16,51,56]. Two of the five studies used the Hopkins Verbal Learning Test (HVLT), a popular memory test used to measure delayed verbal memory. Both studies revealed iron supplementation to have no significant effect on memory and recall scores [50,52].

#### 3.6.4. School Performance

Four studies investigated the effects of iron supplementation on school performance [16,52,54,55]. All studies demonstrated a positive effect of iron treatment on the various measures of school performance. One study reported improved scholastic performance test scores measured through a math test [16]. Two other studies examined the effects of iron supplementation on school performance, accounting for baseline anemia status [54,55]. Iron treatment revealed a positive effect on learning in the anemic group for math, biology, social science, and language [54]. Improvement in scores was also seen in the non-anemic supplemented group for math and biology; however, no statistical analyses were reported [54].

Soemantri et al. reported an overall improvement in achievement scores in the iron supplemented group as compared with placebo. When examined by anemia status at baseline, among the anemic cases, the adjusted mean difference in scores of iron-treated children (mean difference = 3.64) was significantly different from the placebo-treated children (mean difference = −0.67). Conversely, among the non-anemic cases, there were no significant differences in changes between iron (mean difference = −0.29) and placebo (mean difference = 0.28) treated children [55].

One study reported that the iron group’s school performance was significantly better than the placebo group prior to treatment (*p* < 0.04) [52]. Since participants were randomly assigned to the two groups, the authors interpreted this result as a Type 1 error, and no further analyses were reported. Despite this, the study reported a positive association between ferritin change and post-treatment reading span, controlling for pre-treatment reading span, while Hb change was not significantly correlated [52].

#### 3.6.5. Other Measures of Cognitive Performance

One study reported an additional measure of cognitive performance and concluded that there were no significant relationships between iron supplementation and the Stroop task (a measure of interference and processing speed) or the visual search task (a measure of perceptual speed) [52].

### 3.7. The Effects of Iron Plus Additional Micronutrients Supplementation Interventions on Dimensions of Academic Performance and Cognitive Function

Six studies reported on the effects of iron in combination with additional micronutrient supplementation interventions on cognitive performance [13,57,58,59,60,61]. The iron doses in these supplements ranged between 2.5 mg of iron and 36 mg of iron daily, and intervention duration ranged from 4 weeks to one year.

#### 3.7.1. Intelligence

Five studies examined the effects of iron with additional micronutrient supplementation on intelligence [13,58,59,60,61]. Of these, two studies found that supplementation did not affect intelligence [59,61]. Two studies showed mixed results [13,60]; one of which showed that supplementation did not have a significant effect on RPM, verbal intelligence, or the Matrix Analogies Test (MAT) for non-verbal reasoning abilities [60]. However, there was a significant gain of 3.7 points on non-verbal intelligence between the placebo group and 100% recommended dietary allowance (RDA) supplement group, which was not evidenced in either the 50 or 200% RDA supplement groups. This was primarily due to gains in object assembly, coding, and picture arrangement. Supplementation only produced a significant effect over the placebo group for 3 of 13 components (comprehension, battery score, and reading) on a standardized statewide test, the Comprehensive Test of Basic Skills (CTBS) [60].

The second study with mixed results found that experimental subjects showed larger test score improvements than controls [13]. When comparing each group to the control group, the daily iron-folic acid (IFA) and twice-weekly IFA groups scored significantly higher than the control in all four cognitive function tests (digit span, maze test, visual memory test, and clerical task) and once-weekly IFA scored significantly higher in only two of the four tests. Overall, daily IFA and twice-weekly IFA showed marked improvements in most tests, while once-weekly IFA consistently showed less improvement in cognitive test scores [13].

One study found that there was a positive difference in IQ scores between the supplement and placebo group for those with low baseline ferritin levels (<12 ng/mL) [58]. The IQ change following treatment was not statistically significantly different between the groups for participants with moderate ferritin levels (12–20 ng/mL). Among participants with high baseline ferritin levels (>20 ng/mL), the IQ change following treatment was statistically significantly different between the supplement and placebo groups. Overall, there were no significant IQ changes for the sample as a whole, following the intervention period for either the supplement or the placebo group [58].

#### 3.7.2. Other Measures of Cognitive Performance

One study analyzed the effects of iron with additional micronutrient supplementation on 12 different cognitive tasks and found that, following 85 days of daily supplementation, there were no significant differences in unadjusted mean scores for any of the tasks between the treatment and placebo group [57].

## 4. Discussion

This review included 50 studies that examined the association of iron status and anemia with cognition, learning, and school performance and the effects of iron interventions on cognition and school performance in adolescents. Overall, with the limited evidence available, we found iron status and anemia may be associated with school performance in some contexts, yet there was no relationship in other settings. Like previous studies, iron interventions were effective in improving iron status and anemia. Additionally, iron interventions during adolescence may be beneficial for improving school performance, attention and concentration. However, we emphasize that the quality of evidence was low, with nearly all studies evaluated as having either some concerns or a high risk of bias.

### 4.1. Attention and Concentration

Based on the evidence available, our review does not suggest that iron status and/or anemia directly influence, or are associated with, attention and concentration in adolescents. However, supplementation with iron may improve concentration, as three of the four included studies reported a positive effect, although one study was limited to self-reported attention. Food-based interventions may be associated with improvements in attention and concentration, as shown in two of the three studies. Of the studies that found positive effects, one intervention provided a milk-based health drink (with 14 mg of iron twice daily), and the other provided *bhakri* made from iron-biofortified pearl millet (median iron intake/day was 22 mg in the intervention group) [15,46]. The trial that found no significant effect of the intervention provided only 7 mg iron in the form of an orange-flavored drink [44]. As the food-based interventions were drastically different in delivery form, it is challenging to come to definitive conclusions, as other nutritional aspects, such as caloric content and inclusion of other nutrients, may play a role.

### 4.2. Intelligence

We found no evidence to indicate that iron status, anemia, nor dietary iron intake influenced intelligence from the included studies. Furthermore, there was no suggestion that iron supplementation during the adolescent years, with or without additional micronutrients, nor food-based interventions improved intelligence. This may be because intelligence is the most stable psychological trait across the lifespan. Intelligence has a Pearson correlation coefficient of 0.54 from age 11 to 90 years—limiting influence by short-term interventions occurring in adolescence [64,65]. However, nutrition interventions earlier in infancy and childhood have been shown to sustain impacts on adolescent IQ [66].

### 4.3. Memory and Recall 

We found no evidence to suggest that iron status, anemia, food-based interventions, nor iron supplementation influenced memory and recall.

### 4.4. School Performance

There may be evidence to suggest the iron status and anemia influence school performance as reported by 11 of the 16 studies. Additionally, all four of the studies that examined iron supplementation and school performance found positive effects. While one of these studies was unable to report on end-line school performance because of significant baseline differences in school performance between intervention and control groups, a positive association between ferritin change and post-treatment reading span, while controlling for pre-treatment reading span, was observed [52]. No significant association was found in this study for Hb change; however, the duration of intervention was only eight weeks—shorter than the 90–120 days necessary for red blood cell turnover [2].

Both studies that reported on dietary iron intake and school performance had a poor-quality rating. Thus, while the two trials showed a positive association, the quality rating and the small number of studies do not suggest there to be sufficient evidence to support an association between increased dietary iron intake and improved school performance. Additionally, from the studies included in our review, there is no evidence that food-based interventions to increase dietary iron intake improve school performance. A study with a sound design (i.e., randomization and a control group) would be required to determine how provision of iron rich foods, potentially through school meals or food-based interventions, could affect learning. It would be necessary to control for confounders, including baseline anemia status, dietary iron intake, baseline school performance, as well as household factors that influence dietary intake and school performance. This would be of greatest relevance in low- and middle-income countries where anemia and iron insufficiency in the diet are of concern. The duration of the study should account for the time to see improvements in anemia and/or iron status with the selected intervention, as well as the subsequent length of time to see improvements in dimensions of learning being assessed.

### 4.5. Comparison with Other Literature

Overall, our findings varied in agreement with previously published reviews on the topic. Similar to our review, yet with greater strength of evidence, Falkingham et al. concluded that there was a positive effect of iron supplementation on attention and concentration in children >6 years of age, regardless of baseline iron status, and that there was no effect of iron supplementation on memory [6]. Low et al. also reported improved attention and concentration measures following iron supplementation in children aged 3–15 years [8]. Our review is also in agreement with Khor et al., who stated there is a lack of consistent data regarding the impact of micronutrient supplementation on the intelligence of children aged 5–15 years in developing countries [67].

Most markedly, our review, which focused primarily on adolescents 10–19 years of age, differed from the previously published literature on intelligence. For example, Lam et al., whose review included children and adolescents aged 4–15 years, found there to be a significant positive effect of micronutrient supplementation on the fluid intelligence of micronutrient-deficient children [68]. Moreover, Low et al. found that iron supplementation improved global cognitive scores (standardized mean difference = 0.50; 95% CI: 0.11–0.90, *p* = 0.01) and intelligence (mean difference = 4.55; 95% CI 0.16–8.94, *p* = 0.04) among anemic children [8]. Falkingham et al. reiterated this with their findings of iron supplementation improving IQ in anemic children; however, this outcome did not extend to non-anemic children. No effect was found for iron supplementation on psychomotor skills or school achievement [6]. A cognitive improvement from iron supplementation was also noted in a review by Hermoso et al. of anemic and non-anemic infants and children, up to the age of 18 [7]. Here, we were looking at iron interventions in adolescence; however, for adolescents who may have been at risk of inadequate iron intake in their early childhood years, poor outcomes in cognitive function and learning maybe due to earlier cognitive development, which may be difficult to reverse with an intervention initiated during adolescence.

Finkelstein et al. reviewed three studies and found that, in persons aged 12–45 years, iron-biofortified crops improved overall performance in cognitive tasks assessing attention (mean difference in reaction time: −0.22; 95 % CI −0.32, −0.12) and memory (mean difference in reaction time: −0.42; 95 % CI −0.57, −0.27) compared with conventional crops, despite not having a statistically significant effect on ID or anemia [69]. Khor et al. also reported a consistent beneficial effect of micronutrient supplementation on short-term memory [67].

In these reviews, data on adolescents specifically were not presented. Our findings are aligned with more recent reviews on early childhood development, which has been well studied and documented, with anemic children showing poorer cognition and school achievement than non-anemic children [70]. Randomized controlled trials in anemic children >2 years of age were previously shown to have a clear benefit of iron supplementation on cognition [8,70,71]; yet recent reviews show lack of evidence of iron supplementation for short or long term cognitive development [8,71]. Iron supplementation in adolescence showed benefits on academic performance in the limited intervention studies available. However, the same quality and quantity of evidence is not available for the adolescent age group as for younger children, highlighting the focus of previous intervention and research.

### 4.6. Limitations

The major limitation of this review is that a meta-analysis was not possible, hindered by different outcome measures across studies and diversity in the ages sampled. Based on the evidence reviewed, it is difficult to draw firm conclusions about the effects of iron-based interventions for adolescents and iron/anemia status on cognition and school performance. Overall, several included intervention studies were judged to be at high risk for bias, or of poor quality for cross-sectional studies. While our review included studies from a wide range of countries, both high income and low income, there was a large amount of heterogeneity in measures of school performance, limiting the comparability among the included studies. Although global initiatives are underway to strengthen the assessment of learning and education systems [72,73], there are no universal tests of school performance widely implemented.

There was a vast array of tests used to measure intelligence and cognitive performance, limiting comparability among studies. Moreover, due to the quality of some of the papers included, several of the tests used were not adequately described, further limiting comparison. It should also be noted that no intervention included in this review was longer than 14 months, with the majority being less than one year. These shorter trials, in turn, may not be of sufficient duration to influence cognitive or school performance as iron status during learning may be different from iron status at assessment [6]. In contrast, adolescent girls participating in weekly iron and folic acid supplementation (WIFAS) programs could be consuming supplements intermittently for at least 3–5 years, depending on their years of school enrollment and attendance [74]. Several countries have shown increasing interest through multi-sectoral initiatives to address adolescent nutrition, including efforts to scale WIFAS for anemia reduction [75,76] The potential benefits of these programs on student learning are motivating for teachers and the education sector [77].

Many of the studies included did not account for potentially confounding variables that are known to influence cognitive and academic performance. Variables of note include socioeconomic status, a well-established central determinant of academic performance and cognitive ability [78,79]; quality of schooling; and family characteristics; parents’ education level which could be included and measured in randomized studies and others that could potentially be of interest where possible would be attitudes towards school; and an individual’s motivation, behavior, and aptitude [79,80].

### 4.7. Program Implications

Currently, the WHO has guidelines for intermittent and daily iron supplementation that target adolescents. In areas where anemia is highly prevalent (≥40%), menstruating adult women, adolescent girls, and school-aged children are recommended to consume 30–60 mg of elemental iron daily for three consecutive months in a year [81,82]. In areas where the prevalence of anemia is ≥20%, menstruating adult women and adolescent girls are recommended to take WIFAS containing 60 mg elemental iron and 2.8 mg folic acid once weekly for three consecutive months, followed by a period of three months with no supplements or as aligned with the school semester [83]. The inclusion of folic acid in WIFAS may protect against neural tube defects, should a woman or adolescent girl have an unplanned pregnancy [84,85]; however, this requires continuous consumption on a once weekly basis.

Schools are a key platform for reaching a larger number of adolescent girls, as adolescents are the least likely to access health systems for preventative services. As schools are often the primary delivery platform, and teachers and ministries of educations are key partners in WIFAS for anemia reduction as part of adolescent nutrition programs, there is keen interest in the potential impacts of WIFAS on cognition, learning, and school performance [77,86]. Defining the academic and cognitive benefits of WIFAS is of keen interest to the teachers and schools who invest their time as partners in the delivery and reporting of this health intervention. Out-of-school girls, however, will miss out on the long-term nutritional, health, and economic benefits of educational attainment and potential anemia reduction that could occur from accessing school-based supplementation programs. Thus, additional platforms are needed for vulnerable groups.

### 4.8. Conclusions and Future Research

It has been established that supplementation with iron, alone or combined with other micronutrients [87,88,89]; iron-containing micronutrient powders for point-of-use fortification [90]; and food fortification with multiple micronutrients [91] are effective interventions for increasing Hb concentrations and reducing the risk of anemia. However, more high-quality research is needed to draw firm conclusions on the relationship between iron status and anemia and cognitive and academic performance in adolescents. Moreover, the potential effects of iron interventions need to be elucidated with rigorous research if ministries of health and education are to adopt such programming.

One focus should be on assessing longer-term impacts of exposure to iron interventions, such as WIFAS delivered yearly in schools. Researchers and program implementers could implement measures that allow for comparisons across studies that are sensitive to food and nutrient interventions [92]. This, in turn, could allow for meta-analyses and the potential determination of effect sizes and clinical significance. However, this can be challenging due to the sheer number of tests available, the need for education systems to consider local needs, as well as cultural and language differences. Tests that are less susceptible to these differences, such as visual tests, are not the most sensitive to change, and verbal tests, which are sensitive to change, are susceptible to language and cultural differences [92]. Thus, guidance from and collaboration with the education sector is needed to support nutrition researchers in testing and measuring cognitive and academic performance. Moreover, it is pertinent that researchers record and account for confounding variables known to influence cognition and academic performance. Additional emphasis should be placed on accounting for baseline iron status and anemia, using internationally recognized cut-offs with the appropriate corrections [93], and other dietary variables that could influence cognitive and academic performance. The evidence base could benefit from additional cluster randomized trials which assess the effects of iron-containing interventions that not only have proven efficacy for anemia reduction but can also be effectively delivered to reach adolescents in collaboration with schools—thus, enabling the measurement of nutritional, cognitive, and academic outcomes. As investments to improve adolescent nutrition in low- and middle-income countries increase, priority also needs to be given to addressing the research gaps in adolescent nutrition to provide decision makers with high-quality data.

## Figures and Tables

**Table 1:** Description and results of cross-sectional studies examining iron status or anemia and dimensions of academic performance and cognitive function.

Author (Year, Country)	Study Design	Study Population ^1^	Exposure or Nutrition/Anemia Status	Learning/Cognition Outcome Assessed and Measure	Key Results ^2^
Abalkhail et al. (2002, Saudi Arabia) [18]	Cross-sectional study	*n* = 800 school children aged 9–21 y Age: 14 ± 2.6 y	Prevalence of anemia was assessed using Hb estimated by Refletron (Boehringer Mannheim).	School grades were classified according to the national school grading classification.	Higher percentage of anemia among students with marks < 70% (fail/pass) than students with good, very good or excellent grades (no statistical analyses).
Anuar Zaini et al. (2005, Malaysia) [19]	Cross-sectional study	*n* = 1405 standard four primary students, aged 9–10 y Age: 9.7 ± 0.5 y (68% were 10 y)	Prevalence of anemia from finger-prick blood samples with a HemoCue^®^.	School grades in Malay language (comprehension and written), math, English, and science. RCPM for intelligence.	Severe anemics had higher scores in Malay language comprehension and writing, math, and English; lower science scores and RCPM. Moderate anemics had lower science scores and RCPM (no statistical analyses).
Aquilani et al. (2011, Italy) [20]	Cross-sectional study	*n* = 48 high school girls aged 14–15 y Age: 14.6 ± 0.7 y	Daily iron intake (mg) was assessed by a student-kept weighted 7 day food record and analysis was conducted using a computer system designed by the research group.	School achievement was assessed by mid-year curriculum performance in written math, oral math, and written Italian.	Students with satisfactory school performance had higher iron intakes than those with unsatisfactory scores **. Iron intake was significantly positively correlated with written math (*r* = 0.43 ***), oral math (*r* = 0.40 **) and written Italian (*r* = 0.37 *).
Cai and Yan (1990, China) [21]	Cross-sectional study	*n* = 58/478 middle school students aged 13–15 y	Prevalence of IDA was assessed using a 5 mL venous blood sample for Hb, ferritin, and free erythrocyte porphyrin.	IQ was tested using the Bourden–Wisconsin test. School marks in Chinese, math, and English class were taken from school records.	No significant difference in scores for verbal IQ, performance IQ, total IQ, or school marks by subject for students with IDA compared with those without IDA.
Carruyo-Vizcaíno et al. (1995, Venezuela) [22]	Cross-sectional study	*n* = 213 middle-class adolescents ages 12–17 y Age: 13.8 ± 1.3 y	Prevalence of ID and anemia was determined using a CBC, SI, TIBC, TS, and ferritin.	IRA was the ratio between the number of subjects approved over the total number of subjects taken. The final GPA from grades of each subject from three periods of the school year	IRA scores positively correlated with ferritin levels < 20 ug/L (*r* = 0.411 **); negatively correlated with Hb, iron, and ferritin in the total population (not shown); anemic males scored worse than non-anemic males * and all non-anemic adolescents **; no differences in average scores for any other blood parameters. Hb in females was negatively correlated with GPA; no differences in final GPA were found for any blood parameters.
Dissanayake et al. (2009, Sri Lanka) [23]	Cross-sectional study	*n* = 188 Sinhalese students age 13–15 y	Prevalence of ID and IDA was assessed by Hb, determined by the indirect cyanmethemoglobin method, and ferritin.	RPM for intelligence. School marks in science, math, social science, and Sinhala language, and total marks.	No significant relationship was observed between IQ or school performance and iron status or severity of ID.
El Hioui et al. (2012, Morocco) [24]	Cross-sectional study	*n* = 259 primary school children aged 6–16 y Age: 10.2 ± 2.48 y	Prevalence of IDA and anemia was assessed by CBC and ferritin.	RPM for intelligence. School achievement was assessed by the students’ scores in math GPA, cumulative GPA, and rank.	More anemic children had an intellectual deficit *; RPM performance related to Hb level ***. Ferritin was correlated with math (*r* = 0.5 *) and cumulative GPA (*r* = 0.37 *); math GPA was related to Hb level *; iron status related to school achievement ****.
Goudarzi et al. (2008, Iran) [25]	Cross-sectional study	*n* = 540 students aged 11–17 y Age: 14.9 ± 1.2 y	Prevalence of ID was assessed by SI, TIBC, and ferritin.	RPM for intelligence.	No significant difference in IQ scores or IQ classification among students with ID, IDA or normal iron status.
Halliday et al. (2012, Kenya) [17]	Cross-sectional RCT baseline analyses	*n* = 2400 students aged 5–18 y Age: 10.3 y	Prevalence of anemia was assessed using a portable hemoglobinometer.	Attention was assessed by pencil-tap test and the code transmission test. RPM for non-verbal reasoning.	Anemia status was not associated with attention, literacy, non-verbal reasoning, comprehension, or numeracy skills.
Halterman et al. (2001, USA) [26]	Cross-sectional study	*n* = 5398 children aged 6–16 y (61.3% were 6–11 y, 38.7% were 12–16 y)	Prevalence of ID and IDA was determined by TS, ferritin, erythrocyte protoporphyrin, and Hb.	WISCR: verbal component (digit span) and performance examination (block design). WRAT: math and reading components.	For all categories, scores lowered with diminishing iron status. IDA and ID did not score differently than normal status for reading and digit span (ns). For reading, block design, and digit span the % scoring below average did not differ by iron status. ID was not at increased odds of scoring below average for reading, block design, or digit span (ns) but IDA scored lower than children with normal status *. IDA and ID had lower math scores * and had higher risk of scoring below average (OR 2.3; 95% CI: 1.1,4.4).
Hutchinson et al. (1997, Jamaica) [27]	Cross-sectional study	*n* = 800 rural students in grade 5 aged 9–13 y Age: 10.8 ± 0.6 y	Prevalence of anemia was assessed by portable hemoglobinometer. Samples were obtained from 769 children.	WRAT: reading, spelling and math subtests.	Hb was significantly positively correlated with reading and spelling scores but not correlated with math scores.
Ivanovic et al. (2004, Chile) [28]	Cross-sectional study	*n* = 4509 students ages 5–22 y Age: 10.4 ± 3.5 y	Daily iron intake (% of adequacy) from 24 h dietary recall data by individual interviews.	School achievement was evaluated through standard Spanish-language and math achievement tests designed for the study.	Iron intake (% daily value) was correlated with scholastic achievement for the whole sample (*r* = 0.065 ***). By grade, this positive correlation was only significant in grade 4 high school students (*r* = 0.142 *). NS for grade 1 high school, grade 6 or 8.
Ji et al. (2017, China) [29]	Cross-sectional study	*n* = 428 elementary school students aged 11–14 y Age: 12.0 ± 0.4 y	Prevalence of ID from Hb and SI.	CNB was used for performance accuracy and speed in four neurobehavioral domains. Chinese version of the WISCR was used to measure intelligence.	Only one difference in mean raw CNB scores was found * which was ns after adjustment. ID had longer reaction times on tests of mental flexibility and capacity for abstraction and the test of special processing ability *. High SI had slower speed on tests of spatial processing ability * and had decreased abstraction ability and mental flexibility *. Iron status was associated with the full-scale IQ score (ns).
Kharat and Waghmare (2015, India) [30]	Cross-sectional study	*n* = 74 adolescent girls aged 18–19 y	Prevalence of anemia was assessed by Hb concentration, tested by the cyanmethemoglobin method.	Cognitive performance was assessed with P300 using an odd ball paradigm with an RMSEMG EP II machine.	Anemic group had delayed P300 latencies as compared with the control group ****. The P300 amplitudes were larger in the girls in the control group than the anemic group *.
Masalha et al. (2008, Israel) [31]	Cross-sectional study	*n* = 67 fourth, fifth, and sixth graders ages 9–11 y.	Prevalence of anemia was assessed using venous blood was used.	Academic Achievement Index was calculated as the ratio of all marks achieved of all approved courses over the total. Low achievement was classified as scores < 80%.	Of the 14 children with anemia, 6 had low academic achievement scores (42.9%). (no statistical analyses reported.)
More et al. (2013, India) [32]	Cross-sectional study	*n* = 87 girl aged 12−15 y studying in sixth to ninth standard	Screening for anemia and ID was performed by CBC and ferritin.	School achievement was assessed by math score from the final term exam on report cards. Multicomponent Test for verbal learning, memory, and attention; PGI test; and Bhatia battery performance test.	Scholastic performance, IQ, and scores of mental balance, attention and concentration, verbal memory, and recognition were decreased in iron-deficient girls, both anemic and non-anemic, as compared with the non-iron-deficient girls *.
Nagalakshmi et al. (2015, India) [33]	Cross-sectional study	*n* = 60 rural school children aged 9–12 y Age: 10.4 ± 1.1 y	Hb level was assessed using Sahli’s acid hematin method.	Visual reaction time; whole-body reaction time, and MMSE.	Whole-body reaction time was negatively correlated with Hb ***. Visual reaction time and MMSE were negatively correlated with Hb (ns).
Nemati et al. (2005, Iran) [34]	Cross-sectional study	*n* = 170 adolescent girls Age: 12 y	Prevalence of IDA and anemia from venous blood samples. Measured Hb, hematocrit, MCV, TIBC, and ferritin.	“Educational progression including average test score of base class primary school for schoolgirls”. Test scores (/20) were classified as low (10–15) and high (15.1–20).	Anemics had lower test scores than those without anemia *. IDA had significantly lower test scores than those without IDA *. ID did not have significantly lower test scores than those without ID. Hb was correlated with average test score (*r* = 0.171 *). Ferritin was not significantly correlated.
Olson et al. (2009, Philippines) [35]	Cross-sectional study	*n* = 322 rural school students aged 7–18 y Age: 12.1 (95% CI: 11.7,12.4) y	Prevalence of IDA and anemia from a CBC by hematology analyzer on venous blood samples. Ferritin, sTfR were also measured for iron status.	WRAML, verbal fluency, and PNIT.	Students with IDA and NIDA had lower non-verbal intelligence scores than students with no anemia **. After adjustment, anemia status showed no effect on WRAML learning index, but children with NIDA scored worse than children without anemia on the verbal memory component *. Anemia status, regardless of type, had no significant effect after adjustment on verbal fluency.
Ortega et al. (1993, Spain) [36]	Cross-sectional study	*n* = 64 middle-class adolescents aged 15–18 y Age: 15.9 ± 0.8 y	Iron intake was quantified using the 5 day “food consumption registration” technique. Fasting venous blood samples were used for a CBC measured using a Coulter S analyzer Plus. SI was also measured.	Spanish TEA for verbal, reasoning, and calculus. IQ percentile (IQ < or > 100) is calculated from total scores. The attention test consisted of clearly crossing out all the letters that were accompanied by two apostrophes and the hits, errors, omissions, and speed were recorded. School grades for Latin, Spanish language, foreign language, geography, religion-ethics, math, physics-chemistry, physical education, and technical-professional activities were obtained.	In girls, ID was associated with lower scores for verbal, calculus, school aptitude, and IQ *; higher IQ had higher Hb *; iron status was not associated with school grades. In boys, ID was associated with lower factor scores for verbal, reasoning, school aptitude, attention speed, grades in physics and chemistry *; ferritin was positively associated with IQ percentile *. Overall, Hb was associated with calculus score (*r* = 0.2905 *), but not attention, verbal scores, reasoning scores, or overall TEA global scores. Ferritin was associated with attention hits (*r* = 0.3434 *) and speed (*r* = 0.3989 *). Iron intake was negatively associated with attention hits (*r* = −0.2874 *) but not IQ percentile. SI was associated with none of the above scores.
Sen and Kanani (2006, India) [37]	Cross-sectional study	*n* = 350 low-income adolescent girls aged 9–14 y	Prevalence of anemia was measured with Hb by the cyanmethemoglobin method.	Gujarati version of WISC: digit span test for short-term memory, maze test for visual–motor coordination, Clerical task for concentration and ability to discriminate, and visual memory test for short-term memory.	Girls with anemia performed worse on the digit span test and visual memory tests in both 9–11 and 12–14 age ranges *. No difference in performance on the maze test or clerical task by anemia statuses.
SoonMyung et al. (2004, Korea) [38]	Cross-sectional study	*n* = 193 adolescent girls aged 11–14 y Age: 12.6 ± 0.6 y	Prevalence of anemia through Hb was measured using an Automatic Blood Cell Counter. SI, TIBC, and ferritin were also measured.	Questionnaire regarding clinical symptoms of anemia was administered. Decreased ability to concentrate and poor memory were measured using Likert-type scales.	Hb and ferritin were not significantly correlated with decreased ability to concentrate and poor memory.
Teni et al. (2017, Ethiopia) [39]	Cross-sectional study	*n* = 442 adolescent girls aged 10–19 y Age: 14.2 ±1.7 y	Prevalence of anemia measured by the HemoCue (Hb 301) system.	Average scores in the school were obtained from the school records.	Anemic girls were more likely to show low academic performance, compared with non-anemic girls (AOR = 1.7; 95% CI: 1.2, 2.7 *). More anemic girls had academic performance below the mean compared with non-anemic girls (71.1 vs. 64.5%) (no statistics analyses).
Thalanjeri et al. (2016, India) [40]	Cross-sectional study	*n* = 30 school going children both males and females between the ages of 9 and 13 y	Prevalence of anemia was assessed through venous blood was collected for a CBC using a semi-auto hematology analyzer.	Visual memory test and RPM.	RPM scores were lower in anemic children as compared with non-anemic children ***. No significant correlation between Hb and the visual memory test.
Walker et al. (1998, Jamaica) [41]	Cross-sectional study	*n* = 452 adolescent girls aged 13–14 y in grade 8	Prevalence of anemia using Hb measured by an automated method on a Cell Dyn 700 cell counter.	School achievement using the WRAT for spelling, reading, and arithmetic. Scores on the test were converted to grade levels.	Anemia was associated with lower achievement levels in reading and spelling **.
Webb and Oski (1973, USA) [42]	Cross-sectional study	*n* = 193 students ages 12–14 y in a junior high school low SES black community	Prevalence of anemia assessed by CBC using the Coulter Counter, Model S.	School achievement using the composite score of the Iowa Tests of Basic Skills.	Anemic subjects differed from non-anemic subjects in composite scores achieved *. Anemic girls aged 12 y scored better than non-anemic girls. All other anemic subjects scored worse than non-anemic subjects.

CBC, complete blood count; CNB, Penn Computerized Neurocognitive Battery; GPA, grade point average; Hb, hemoglobin; ID, iron deficiency; IDA, iron-deficiency anemia; IQ, intelligence quotient; IRA, Academic Performance Index; MCV, mean corpuscular volume; MMSE, mini-mental state examination; OR, odds ratio; PNIT, Philippines non-verbal intelligence test; RCPM, Raven’s Colored Progressive Matrices; RPM, Raven’s Progressive Matrices; SI, serum iron; sTfR, soluble transferrin receptor; TEA, Test of Educational Ability; TIBC, total iron-binding capacity; TS, transferrin saturation; WISCR, Wechsler Intelligence Scale for Children-Revised; WRAML, Wide- Range Assessment of Memory and Learning; WRAT, Wide-Range Achievement Test-Revised. ^1^ Age is presented as the mean age ± SD unless otherwise stated. ^2^ Effect estimates, when available, are presented with associated significance value (ns *p* > 0.05, * *p* < 0.05, ** *p* < 0.01, *** *p* < 0.001, and **** *p* < 0.0001).

**Table 2:** Description and results of iron-containing intervention studies examining dimensions of academic performance and cognitive function.

Author (Year, Country)	Study Design	Population ^1^	Exposure	Learning and Cognition Outcome Assessment Method	Key Results ^2^
**Food-based interventions**					
Kalaichelvi (2016, India) [43]	Randomized, controlled trial (Dissertation)	*n* = 240 adolescent girls with anemia in the age group of 12–15 y	8 weeks; intervention given 5 days a week (Monday to Friday): 13.14 mg of iron from a nutritional ball (60 g roasted rice flakes and 40 g of jaggery) and alma fruit powder (24 mg vitamin C); Regular nutritional practices.	Malin’s intelligence scale for Indian children for verbal response, general information, arithmetic, similarities and digit span and in performance, picture completion, object assembly, coding, and maze.	Intelligence scores increased in the treatment group *, no change in control group; post-test scores were higher in anemic girls in treatment group than anemic controls ***; positive correlation between Hb level and intelligence score (*r* = 0.84 **).
Karkada et al. (2019, India) [12]	Quasi-experimental trial	*n* = 60 semi-rural adolescent girls aged 11–17 y	90 days; twice-daily intervention: 1–2 tablespoons of Ragi powder containing 3.7–6.8 mg iron per 100 g. (~0.58–2.12 mg iron per day from the powder ^3^); Usual diet, no intervention.	The scholastic performance was determined by exam percentage scores before data collection.	No statistically significant changes in school performance were found following the intervention.
Khan et al. (2004, Bangladesh) [44]	Randomized, double-blind, placebo-controlled trial	*n* = 317/1268 adolescent girls aged 11–14 y selected for cognitive testing and *n* = 697/1268 for school achievement test Age: 12.0 y	12 months; each subject received one 200 mL serving of the constituted beverage for six days a week (Saturday to Thursday). Orange-flavored micronutrient-fortified powdered beverage mix containing 13 micronutrients (7.0 mg of iron); Placebo: non-fortified, orange-flavored mix.	RCPM and tests of verbal fluency, visual search, and free recall. WRAT containing sections of spelling and arithmetic.	After 12 mo., intervention group scored higher in spelling ** and math ** than placebo. In non-anemic girls, a negative trend in visual search test scores was seen in the intervention compared with placebo *; no difference in anemic. No difference in between groups for free recall, RCPM and verbal fluency; anemic supplemented girls trended better than anemic placebo girls in RCPM and verbal fluency (ns).
Muthayya et al. (2012, India) [45]	Randomized, double-blind, controlled, school feeding trial	*n* = 401 urban and rural primary school children aged 6–15 y Age: 10.4 y	7 months; daily lunch 6 days/week: Wheat-based lunch meal fortified with 6 mg of iron as NaFeEDTA (11.2 ± 0.7 mg iron/meal); Identical wheat-based meal with no fortified iron (5.1 ± 0.6 mg iron/meal).	Cognitive tests used were the Atlantis, Kohs block design, word order, pattern reasoning, verbal fluency, and coding WISC-III.	No effect of treatment on cognitive performance after adjusting for baseline scores. No significant interaction effect of treatment for gender, grouping, ferritin, or body iron store.
Scott et al. (2018, India) [46]	Double-blind, randomized, intervention study	*n* = 140 rural students (aged 12–16 y) Age: 13.7 y	6 months; 200–300 g (dry) pearl millet/d in the form of bhakri during lunch and dinner: Iron-biofortified pearl millet (86.3 ppm iron). Iron intake ^4^ was 22.0 (18.4, 25.2) mg/d; Control pearl millet (21.8 ppm iron switched to 52.1 ppm after 4 mo.) Iron intake ^4^ was 9.1 (7.7, 10.3) mg/d.	Five cognitive/behavioral tasks (3 attention tasks and 2 memory tasks) were administered on laptop computers: SRT, GNG for sustained attention and speed of simple attentional capture, ANT, CFE, and CRT.	The consumption of biofortified pearl millet resulted in greater improvement in attention (SRT, GNG, and ANT) and memory (CFE and CRT **). Reaction time decreased twice as much from 0 to 6 mo. in those consuming biofortified on attention tasks **.
Solon et al. (2003, Philippines) [47]	Randomized, double-blind, placebo-controlled field efficacy trial	*n* = 808 children in grades 1–6 Age: 9.95 y	16 weeks; 200 mL serving of either fortified or non-fortified beverage twice each school day: Fortified beverage for which a single serving contained iron (4.8 mg) and 10 additional micronutrients; Placebo unfortified beverage. 50% were randomized to receive 400 mg albendazole.	Primary Mental Abilities Test for Filipino Children for three basic mental abilities: verbal, non-verbal, and quantitative.	Fortified beverage showed no significant effect on change in total cognitive scores for all subjects. Among moderate to severe anemics at baseline, children receiving the fortified beverage showed improvement in changes in non-verbal ability score *.
Sorensen et al. (2015, Denmark) [14]	Cluster-randomized trial with cross-over design (baseline analysis only)	*n* = 726 Danish third- and fourth-grade children ages 8–11 y Age: 10.0 ± 0.6 y	3 months cross-over (6 months total) on school days: Prepared ad libitum lunch and a mid-morning and afternoon snack in line with the Nordic recommendations for a healthy dietary intake. Iron intake in intervention period was 9.2 (7.9, 10.9) ^4^ mg/d; Control: usual lunch habits. Iron intake in the control period was 8.5 (7.4, 10.0) mg/d.	At baseline and at the end of each study period (3 mo and 6 mo.), three tests related to concentration and school performance were administered: d2 Test of attention, the Sentence Reading Test 2, and a math test.	Low iron was associated with poor school performance in girls but not boys *. Children with low iron scored worse for attention and concentration ***. Iron stores were not associated with math scores. Girls with low iron had a worse reading speed and lower number of correct sentences ***. Boys with low iron had higher reading speed and correct number of sentences ***. Low iron was associated with a higher % correct in reading and was associated with reading comprehension in both sexes *.
Vazir et al. (2006, India) [15]	Double-blind, placebo-controlled, matched-pair, cluster, randomized feeding trial	*n* = 608 middle-income semi-urban children ages 6–15 y Age: 10.7 y	14 months; beverage was served twice daily: Health drink plus a micronutrient supplement with 14 mg of iron; Placebo formulation of the health drink without the added micronutrient supplement.	Knox Cube Imitation Test and Letter Cancellation Test for attention, Malin’s Intelligence Scale for Indian Children, and PGI Memory Scale. School marks in science, math, and social studies and aggregate marks of quarterly and final annual examinations were used.	Supplementation significantly improved attention and concentration **. Supplementation made no significant improvements on IQ scores, memory scores, or school achievement.
Vinodkumar et al. (2009, India) [48]	Randomized controlled trial	*n* = 162/323 children aged 5–18 y Age: 12.3 y	9 months of school meals fortified with: 10 mg of elemental iron as chelated ferrous sulphate through the multiple micronutrient fortified cooking salt; Iodized salt.	Memory tests were given to children aged 11–18 years.	Memory scores of the experimental group were significantly higher than those of the control group, repeated-measures ANOVA showed a group x time interaction *. Treatment group showed significant improvement in memory scores compared with control group.
**Iron-only supplementation interventions**					
Ballin et al. (1992, Israel) [49]	Double-blind, placebo-controlled prospective study (trial)	*n* = 59 urban, middle-class high school girls aged 16–17 y	2 months; once-daily intervention: 10 mL of iron polystyrene sulfonate adsorbate syrup (105 mg of elemental ferrous iron); Placebo liquid.	Questionnaires were given for information about lassitude, fatigue, the ability to concentrate in school, mood, appetite, and quality of sleep.	Girls who received the iron intervention reported significant improvement in lassitude *, the ability to concentrate in school *, and mood *.
Bruner et al. (1996, USA) [50]	Double-blind, placebo-controlled randomized clinical trial	*n* = 81 high school girls aged 13–18 y	8 weeks; twice-daily intervention: Two 325 mg tablets of ferrous sulphate (260 mg elemental iron daily); Placebo.	Brief Test of Attention for auditory attention; Symbol Digit Modalities Test for visual attention, motor speed, and rapid coding; Visual Search and Attention Test for visual scanning, target detection, and cancellation; HVLT for recall and recognition	Iron treatment had no significant effect on post-intervention Brief Test of Attention, Symbol Digit Modalities Test, Visual Search and Attention Test, or Hopkins Verbal Learning Test scores.
Chellappa and Karunanidhi (2012, India) [51]	Randomized, double-blind, placebo-controlled, intervention trial	*n* = 109 low and middle income 17–19 y adolescent girls Age: 18.4 y	16 weeks; once-daily intervention: Ferrous fumarate (184.6 mg = 60 mg elemental iron); Zinc sulphate (82.4 mg = 30 mg elemental zinc); Ferrous fumarate and zinc sulphate (184.6 mg iron and 82.4 mg zinc); Placebo.	Digit Symbol Substitution Test for mental speed, Digit Vigilance test for sustained attention, Standard Progressive Matrices for abstract reasoning, the Rey Auditory Verbal Learning Test for verbal memory and recognition; Rey Complex Figure Test and PGI Memory Scale for visual memory and recognition.	Iron and FeZn supplementation produced significantly higher adjusted post-test scores for mental speed error component **; visual memory immediate ** and delayed recall * compared with placebo. No intervention improved sustained attention, abstract reasoning, immediate and delayed recall of verbal material, and verbal and visual recognition compared with placebo.
Devaki et al. (2009, India) [16]	Single-center prospective placebo-controlled study	*n* = 120 healthy adolescent girls and boys aged 15–18 y Divided into groups based on iron and anemia status	8 months; once-daily intervention, 6 days a week: ID supplemented: oral IPC with 100 mg elemental iron; IDA supplemented: oral IPC (above); Control supplemented: oral IPC (above); Control placebo.	Cognitive performance was measured using: STM, LTM, RPM, WAIS. Scholastic performance was assessed by a math test.	All groups that received iron supplements had significantly improved test scores for STM, LTM, RPM, WAIS, and scholastic performance compared with the placebo group **.
Lambert et al. (2002, New Zealand) [52]	Randomized, double-blind intervention study	*n* = 116 female high school students aged 12.5–17.9 y with ID Age: 15.2 y	8 weeks; once-daily intervention: Ferrogradumet (Abbott) of 105 mg of elemental iron; Placebo.	HVLT, Stroop task, Visual Search task, and reading span task.	Reading span was positively correlated with ferritin (*r* = 0.250 **). Relationship between ferritin change scores and post-treatment reading span (B = 0.22 **) controlled for pre-treatment reading span. No significant relationship was found for delayed recall and recognition scores on the HVLT, Stroop task, or visual search task and iron treatment.
Rezaeian et al. (2014, Iran) [53]	Blind, controlled, clinical trial study	*n* = 200 female students aged 14–18 y Age: 16.2 ± 1.3 y	16 weeks; twice-weekly supplementation: 50 mg ferrous sulphate tablet; No intervention.	The Toulouse–Piéron test for attention score.	Iron supplementation was associated with a positive increase in attention scores ***.
Soemantri et al. (1989, Indonesia) [54]	Double-blind, randomized clinical trial	*n* = 130 primary school children aged 8.1–11.6 y Age: 10.5 y	3 months of intervention followed by 3 months of no intervention: Ferrous sulphate tablets at a dosage of 10 mg/kg/d = 2 mg elemental iron; Placebo.	RCPM for general intelligence. An educational achievement test in math, biology, social science, and language.	Iron supplementation produced no significant effects on IQ at any time point. Iron treatment had a positive effect on learning in the anemic children in the four subject areas; scores improved in the non-anemic children for math and biology (no statistical analyses).
Soemantri et al. (1985, Indonesia) [55]	Randomized, placebo-controlled trial	*n* = 119 anemic and non-anemic school children. Age: 10.9 y	3 months of intervention: Ferrous sulphate tablets at a dosage of 10 mg/kg/d = 2 mg elemental iron; Placebo.	Educational achievement test in math, biology, social science, and language. The Bourden–Wisconsin test for concentration.	Iron significantly improved adjusted school achievement scores. In anemics, the adjusted score of the iron group was significantly higher than the placebo group; no significant differences in non-anemics. Iron group had a significantly higher increase in concentration scores than placebo.
Umamaheswari et al. (2011, India) [56]	Intervention study	*n* = 100 upper-low SES randomly selected children ages 6–11 y (60% aged 9–11 y)	3 months: Ferrous sulphate tablets: 2 mg/kg body weight; Zinc syrup: 5 mg once daily; Iron tablet + zinc syrup; Control: advised nutritious food.	Intelligence was assessed using the Binet–Kamath scale. Memory was tested using: digit forward, sentence repetition, story recall, picture recall, Benton visual retention test, and Cattell’s retentivity test.	ID children had lower verbal memory, non-verbal memory, and IQ scores than normal controls. After supplementation, ID children showed larger improvement scores for all fields compared with normal controls (no statistical analyses).
**Iron plus additional micronutrients supplementation interventions**					
Haskell et al. (2008, England) [57]	Randomized, double-blind, placebo-controlled, parallel groups trial	*n* = 81 children aged 8–14 y Age: 11.1 y	12 weeks; daily intervention at breakfast: Micronutrient supplement that contained 2.5 mg of iron as ferrous (II) fumarate and 17 other micronutrients; Placebo.	Cognitive battery for the speed and accuracy of attention and aspects of memory (secondary, semantic, and spatial working).	No significant differences were found for any unadjusted mean scores at 12 weeks between the treatment and placebo group following micronutrient supplementation.
Lynn and Harland (1998, England) [58]	Placebo-controlled trial	*n* = 413 comprehensive school students aged 12–16 y Age: 13.1 y	16 weeks; once-daily intervention: Iron supplement tablets containing 17 mg elemental iron with 70 mg ascorbic acid; Placebo.	RPM were used, raw scores on the matrices were transformed to age-standardized percentiles from the test manual and percentiles were transformed to IQs.	Overall, there were no significant changes in IQ following the treatment period for either group. For participants with low and high ferritin levels, following treatment, the gain in IQ points was higher in the treatment group than placebo *.
Nelson et al. (1990, England) [59]	Randomized, double-blind, placebo-controlled trial	*n* = 227 school children aged 7–12 y	4 weeks; once-daily intervention: Vitamin-mineral supplement that contained 15 mg iron + 22 other vitamins and minerals; Placebo.	Children 7–10 y completed the Heim AHlX test of non-verbal intelligence, and children 11–12 y completed the Heim AH4 test of verbal and non-verbal intelligence. All children completed the WISCR digit span and coding tests.	The supplement did not affect intelligence.
Schoenthaler et al. (1991, USA) [60]	Randomized, triple-blind placebo-controlled trial	*n* = 615 8th graders (aged 12–13 y) and 10th graders (aged 15–16 y)	13 weeks; each student took one dose Tues to Thurs with a double dose on Mon and Fri: 50% of RDA for 13 vitamins + 10 minerals (9 mg iron); 100% of RDA for 13 vitamins + 10 minerals (18 mg iron); 200% of RDA for 13 vitamins + 10 minerals (36 mg iron); Placebo.	WISCR, MAT, RT/IT, and CTBS. RPM after one month of supplementation, no retest.	For WISCR, gains in the 100% RDA group vs. the placebo in non-verbal intelligence *, primarily due to gains in object assembly, coding, and picture arrangement. No difference in the 50% nor 200% group and supplementation did not affect verbal intelligence. Treatment only produced an effect over the placebo group for 3/13 components in CTBS: comprehension, battery, and reading *. Treatment did not affect MAT and RPM.
Sen and Kanani (2009, India) [13]	Cluster randomized, control trial	*n* = 161 girls in Standards V–VI aged 9–13 y	One year of interventions: IFA tablets (100 mg elemental iron + 0.5 mg folic acid) taken once weekly; IFA tablets taken twice weekly; IFA tablets taken daily; Control: received nothing.	Gujarati version of WISC: digit span for short-term memory; maze test for visual–motor coordination and speed, and fine motor coordination; visual memory test for short-term memory; and Clerical task for concentration and discrimination.	Experimental subjects showed a higher increase in test scores than controls. Overall, IFA-Daily and IFA-2Wkly showed improvements in most tests, while IFA-1Wkly consistently showed less improvement. Cognitive function scores were higher among those who gained more than 1 g/dL Hb (ns).
Southon et al. (1994, England) [61]	Placebo-controlled intervention trial	*n* = 51 adolescents aged 13–14 y Age: 13.8 y	16 weeks, two capsules per day: 12 mg iron as ferrous sulphate + 16 other micronutrients at 50% of the UK RDA value; Mannitol-based placebo.	WISC—Anglicized Revised Edition.	No treatment effect was observed on either total verbal or total non-verbal test scores in the subjects.

ANOVA, analysis of variance; ANT, Attentional Network Task; CFE, Composite Face Effect; CRT, Cued Recognition Task; CTBS, Comprehensive Test of Basic Skills; FeZn, iron and zinc; GNG, Go/No-Go; HVLT, Hopkins Verbal Learning Test; IFA, iron and folic acid; IPC, iron (III) hydroxide polymaltose complex; IQ, intelligence quotient; LTM, long-term memory; MAT, Matrix Analogies Test; NaFeEDTA, ferric sodium ethylenediaminetetraacetate; PGI, post-graduate institute; RCPM, Raven’s Colored Progressive Matrices; RPM, Raven’s Progressive Matrices; RT/IT, reaction time and inspection time; STM, short-term memory; SRT, simple reaction time; WAIS, Weschler Adult Intelligence Scale; WISC, Wechsler Intelligence Scale for Children; WRAT, Wide-Range Achievement Test. ^1^ Age is presented as the mean age ± SD unless otherwise stated. ^2^ Effect estimates, when available, are presented in parentheses with associated significance value (ns *p* > 0.05, * *p* < 0.05, ** *p* < 0.01, and *** *p* < 0.001). ^3^ Estimated from: 1 tbsp of all-purpose flour = 7.81 g. ^4^ Median (interquartile range).

**Table 3:** Risk of bias summary for randomized iron-containing intervention studies assessing academic outcomes or dimensions of learning.

Study	Randomization	Intervention Deviations	Missing Data	Outcome Measurement	Selection of Reported Results	Overall
**Food-based interventions**						
Kalaichelvi (2016) [43]	L	H	L	H	L	H
Karkada et al. (2019) [12]	L	H	L	U	H	H
Khan et al. (2004) [44]	L	L	L	L	U	S
Muthayya et al. (2012) [45]	L	L	L	L	L	L
Scott et al. (2018) [46]	L	L	L	L	L	L
Solon et al. (2003) [47]	L	L	L	L	L	L
Sorensen et al. (2015) [14]	L	L	L	L	L	L
Vazir et al. (2006) [15]	L	L	L	L	L	L
Vinodkumar et al. (2009) [48]	L	L	L	L	L	L
**Iron-only supplementation interventions**						
Ballin et al. (1992) [49]	H	H	H	H	U	H
Bruner et al. (1996) [50]	L	L	L	U	U	S
Chellappa and Karunanidhi (2012) [51]	L	L	U	L	U	S
Lambert et al. (2002) [52]	L	U	L	L	U	S
Rezaeian et al. (2014) [53]	L	U	L	L	U	S
Soemantri et al. (1989) [54]	L	H	L	H	U	H
Soemantri et al. (1985) [55]	U	U	U	H	U	H
Umamaheswari et al. (2011) [56]	H	H	U	L	H	H
**Iron plus additional micronutrients supplementation interventions**						
Haskell et al. (2008) [57]	L	L	L	L	L	L
Lynn and Harland (1998) [58]	L	L	L	U	H	H
Nelson et al. (1990) [59]	L	U	L	L	U	S
Schoenthaler et al. (1991) [60]	L	U	H	L	U	H
Sen and Kanani (2009) [13]	L	U	U	L	U	S
Southon et al. (1994) [61]	H	U	L	L	L	H

H—high risk of bias; L—low risk of bias; S—some concerns; U—unclear risk of bias Risk of bias domains: bias arising from the randomization process, bias due to deviations from intended interventions, bias due to missing outcome data, bias in measurement of the outcome, and bias in selection of the reported result.

**Table 4:** Risk of bias summary for non-randomized iron intervention studies assessing academic outcomes or dimensions of learning.

Study	Confounding	Participant Selection	Intervention Classification	Intervention Deviations	Missing Data	Measurement of Outcomes	Selection of Reported Results	Overall
**Iron Supplementation Intervention**								
Devaki et al. (2009) [16]	M	L	L	L	L	L	L	M

M—moderate risk of bias; L—low risk of bias risk of bias domains: bias due to confounding, bias in selection of participants into the study, bias in classification of interventions, bias due to deviations from intended interventions, bias due to missing data, bias in measurement of outcomes, and bias in selection of the reported result.

**Table 5:** Study quality assessment using the NHLBI Quality Assessment Tool for observational cohort and cross-sectional studies that examined iron and dimensions of academic performance and learning [11].

Observational Cross-Sectional Studies	1. Clear Research Question	2. Clear Study Population	3. 50% Participation Rate	4. Groups Recruited from the Same Population	5. Sample Size Justification	6. Exposure Assessed Prior to Outcome Measure	7. Sufficient Timeframe to See Effect	8. Different Levels of the Exposure of Interest	9. Exposure Measures and Assessment	10. Repeated Exposure Assessment	11. Outcome Measures	12. Blinding of Outcome Assessors	13. Follow-Up Rate	14. Statistical Analysis	Overall Quality Rating
Abalkhail et al. (2002) [18]	Y	Y	Y	Y	N	N	N	N	Y	N	N	U	Y	N	Poor
Anuar Zaini et al. (2005) [19]	Y	Y	Y	Y	N	N	N	Y	U	N	N	U	U	N	Poor
Aquilani et al. (2011) [20]	Y	Y	U	Y	N	U	U	N	N	N	N	U	Y	N	Poor
Cai et al. (1990) [21]	Y	Y	U	Y	N	N	N	N	U	N	U	Y	U	U	Poor
Carruyo-Vizcaíno et al. (1995) [22]	Y	N	N	Y	N	N	N	Y	Y	N	U	U	U	N	Poor
Dissanayake et al. (2009) [23]	Y	U	Y	Y	N	N	N	Y	Y	N	Y	U	U	N	Poor
El Hioui et al. (2012) [24]	Y	Y	U	U	N	N	N	N	Y	N	Y	U	U	N	Poor
Goudarzi et al. (2008) [25]	Y	Y	U	U	N	N	N	N	U	N	Y	N	U	N	Poor
Halliday et al. (2012) [17]	Y	Y	Y	Y	Y	N	N	N	Y	N	Y	U	U	Y	Unclear
Halterman et al. (2001) [26]	Y	Y	Y	N	N	N	N	N	Y	N	Y	Y	U	Y	Poor
Hutchinson et al. (1997) [27]	Y	Y	Y	Y	Y	N	N	Y	Y	N	Y	N	U	Y	Unclear
Ivanovic et al. (2004) [28]	Y	Y	U	Y	Y	N	N	Y	N	N	Y	U	U	N	Poor
Ji et al. (2017) [29]	Y	Y	N	Y	N	N	N	Y	Y	N	Y	U	U	Y	Poor
Kharat et al. (2015) [30]	Y	N	U	Y	N	N	N	N	Y	N	Y	U	U	N	Poor
Masalha et al. (2008) [31]	Y	N	Y	Y	N	N	N	N	N	N	N	U	U	N	Poor
More et al. (2013) [32]	Y	Y	Y	Y	N	N	N	N	Y	N	Y	Y	U	N	Poor
Nagalakshmi et al. (2015) [33]	Y	N	U	Y	N	N	N	Y	N	N	Y	U	U	N	Poor
Nemati et al. (2007) [34]	Y	N	U	Y	N	N	N	Y	Y	N	N	U	U	N	Poor
Olson et al. (2009) [35]	Y	Y	Y	Y	N	N	N	Y	Y	N	Y	U	U	Y	Poor
Ortega et al. (1993) [36]	Y	N	U	U	N	N	N	Y	Y	N	U	U	U	N	Poor
Sen et al. (2006) [37]	Y	N	U	Y	N	N	N	Y	Y	N	Y	U	U	Y	Unclear
SoonMyung et al. (2004) [38]	N	N	U	Y	N	N	N	Y	Y	N	N	U	U	N	Poor
Teni et al. (2017) [39]	Y	Y	U	Y	Y	N	N	N	Y	N	N	U	U	N	Poor
Thalanjeri et al. (2016) [40]	N	N	U	Y	U	N	N	Y	Y	N	Y	U	U	N	Poor
Walker et al. (1998) [41]	Y	Y	N	Y	N	N	N	N	Y	N	Y	U	U	N	Poor
Webb et al. (1973) [42]	Y	N	N	Y	N	N	N	N	Y	N	Y	U	U	N	Poor

## Supplementary Materials

The following supporting information can be downloaded at: https://www.mdpi.com/article/10.3390/nu14010224/s1, Table S1: Medline search strategy for 1946 to 5 December 2019, Table S2: EMBASE search strategy for 1974 to 5 December 2019, Table S3: CINAHL search strategy for 1982 to 5 December 2019, Table S4: PRISMA Checklist.

## References

[1] Lynch S., Pfeiffer C.M., Georgieff M.K., Brittenham G., Fairweather-Tait S., Hurrell R.F., McArdle H.J., Raiten D.J. (2018). Biomarkers of Nutrition for Development (BOND)-Iron Review. J. Nutr..

[2] Gropper S., Smith J. (2013). Advanced Human Nutrition and Metabolism.

[3] Bothwell T., Charlton R., Cook J., Finch C. (1979). Iron Metabolism in Man.

[4] World Health Organization (2017). Global Accelerated Action for the Health of Adolescents (AA-HA!) Implementation Guidance, 2016–2030.

[5] Burrows T., Goldman S., Pursey K., Lim R. (2017). Is There an Association between Dietary Intake and Academic Achievement: A Systematic Review. J. Hum. Nutr. Diet..

[6] Falkingham M., Abdelhamid A., Curtis P., Fairweather-Tait S., Dye L., Hooper L. (2010). The Effects of Oral Iron Supplementation on Cognition in Older Children and Adults: A Systematic Review and Meta-Analysis. Nutr. J..

[7] Hermoso M., Vucic V., Vollhardt C., Arsic A., Roman-Viñas B., Iglesia-Altaba I., Gurinovic M., Koletzko B. (2011). The Effect of Iron on Cognitive Development and Function in Infants, Children and Adolescents: A Systematic Review. Ann. Nutr. Metab..

[8] Low M., Farrell A., Biggs B., Pasricha S. (2013). Effects of Daily Iron Supplementation in Primary-School-Aged Children: Systematic Review and Meta-Analysis of Randomized Controlled Trials. CMAJ Can. Med. Assoc. J. = J. L’Assoc. Med. Can..

[9] Sterne J., Savović J., Page M., Elbers R., Blencowe N., Boutron I., Cates C., Cheng H.-Y., Corbett M., Eldridge S. (2019). RoB 2: A Revised Tool for Assessing Risk of Bias in Randomised Trials. BMJ.

[10] Sterne J., Hernán M., Reeves B., Savović J., Berkman N., Viswanathan M., Henry D., Altman D., Ansari M., Boutron I. (2016). ROBINS-I: A Tool for Assessing Risk of Bias in Non-Randomized Studies of Interventions. BMJ.

[11] National Institute of Health Quality Assessment Tool for Observational Cohort and Cross-Sectional Studies. https://www.nhlbi.nih.gov/health-topics/study-quality-assessment-tools.

[12] Karkada S., Upadhya S., Upadhya S., Bhat G. (2019). Beneficial Effects of *Ragi* (Finger Millet) on Hematological Parameters, Body Mass Index, and Scholastic Performance among Anemic Adolescent High-School Girls (AHSG). Compr. Child Adolesc. Nurs..

[13] Sen A., Kanani S.J. (2009). Impact of Iron-Folic Acid Supplementation on Cognitive Abilities of School Girls in Vadodara. Indian Pediatr..

[14] Sorensen L.B., Damsgaard C.T., Dalskov S.M., Petersen R.A., Egelund N., Dyssegaard C.B., Stark K.D., Andersen R., Tetens I., Astrup A. (2015). Diet-Induced Changes in Iron and n-3 Fatty Acid Status and Associations with Cognitive Performance in 8–11-Year-Old Danish Children: Secondary Analyses of the Optimal Well-Being, Development and Health for Danish Children through a Healthy New Nordic Diet. Br. J. Nutr..

[15] Vazir S., Nagalla B., Thangiah V., Kamasamudram V., Bhattiprolu S. (2006). Effect of Micronutrient Supplement on Health and Nutritional Status of Schoolchildren: Mental Function. Nutrition.

[16] Devaki P.B., Chandra R.K., Geisser P. (2009). Effects of Oral Iron(III) Hydroxide Polymaltose Complex Supplementation on Hemoglobin Increase, Cognitive Function, Affective Behavior and Scholastic Performance of Adolescents with Varying Iron Status: A Single Centre Prospective Placebo Controlled Study. Arzneim.-Forsch./Drug Res..

[17] Halliday K.E., Karanja P., Turner E.L., Okello G., Njagi K., Dubeck M.M., Allen E., Jukes M.C.H., Brooker S.J. (2012). Plasmodium Falciparum, Anaemia and Cognitive and Educational Performance among School Children in an Area of Moderate Malaria Transmission: Baseline Results of a Cluster Randomized Trial on the Coast of Kenya. Trop. Med. Int. Health.

[18] Abalkhail B., Shawky S. (2002). Prevalence of Daily Breakfast Intake, Iron Deficiency Anaemia and Awareness of Being Anaemic among Saudi School Students. Int. J. Food Sci. Nutr..

[19] Anuar Zaini M.Z., Lim C.T., Low W.Y., Harun F. (2005). Effects of Nutritional Status on Academic Performance of Malaysian Primary School Children. Asia-Pac. J. Public Health.

[20] Aquilani R., Maggi L., Parisi U., Ghioni G., Zucchella M., Nardi T., Lombardi P., Covini C., Verri M., Barbieri A. (2011). School Performance Is Associated with Dietary Iron and Zinc Intake in Adolescent Girls. Curr. Top. Nutraceutical Res..

[21] Cai M.Q., Yan W.Y. (1990). Study on Iron Nutritional Status in Adolescence. Biomedical and environmental sciences. BES.

[22] Carruyo-Vizcaíno C., Vizcaíno G., Diez-Ewald M., Arteaga-Vizcaíno M., Torres-Guerra E. (1995). Concentration of Haemoglobin and Nutrients in Middle-Class Adolescents. Relationship to Academic Achievement. Investig. Clín..

[23] Dissanayake D.S., Kumarasiri P.V., Nugegoda D.B., Dissanayake D.M. (2009). The Association of Iron Status with Educational Performance and Intelligence among Adolescents. Ceylon Med. J..

[24] El Hioui M., Azzaoui F.-Z., Ahami A.O.T., Rusinek S., Aboussaleh Y. (2012). Iron Deficiency and Cognitive Function among Moroccan School Children. Nutr. Ther. Metab..

[25] Goudarzi A., Mehrabi M.R., Goudarzi K. (2008). The Effect of Iron Deficiency Anemia on Intelligence Quotient (IQ) in under 17 Years Old Students. Pak. J. Biol. Sci. PJBS.

[26] Halterman J.S., Kaczorowski J.M., Aligne C.A., Auinger P., Szilagyi P.G. (2001). Iron Deficiency and Cognitive Achievement among School-Aged Children and Adolescents in the United States. Pediatrics.

[27] Hutchinson S.E., Powell C.A., Walker S.P., Chang S.M., Grantham-McGregor S.M. (1997). Nutrition, Anaemia, Geohelminth Infection and School Achievement in Rural Jamaican Primary School Children. Eur. J. Clin. Nutr..

[28] Ivanovic D.M., Perez H.T., Olivares M.G., Diaz N.S., Leyton B.D., Ivanovic R.M. (2004). Scholastic Achievement: A Multivariate Analysis of Nutritional, Intellectual, Socioeconomic, Sociocultural, Familial, and Demographic Variables in Chilean School-Age Children. Nutrition.

[29] Ji X., Cui N., Liu J. (2017). Neurocognitive Function Is Associated With Serum Iron Status in Early Adolescents. Biol. Res. Nurs..

[30] Kharat P.S., Waghmare P.P. (2015). Could Anemia Be the Reason for Dysfunctional Cognition?. Int. J. Res. Med. Sci..

[31] Masalha R., Afawi Z., Mahajnah M., Mashal A., Hallak M., Alsaied I., Bolotin A., Ifergan G., Wirguin I. (2008). The Impact of Nutritional Vitamin B12, Folate and Hemoglobin Deficiency on School Performance of Elementary School Children. J. Pediatr. Neurol..

[32] More S., Shivkumar V.B., Gangane N., Shende S. (2013). Effects of Iron Deficiency on Cognitive Function in School Going Adolescent Females in Rural Area of Central India. Anemia.

[33] Nagalakshmi P., Santhosh H., Shobha C. (2015). A Study of Correlation between Hemoglobin Level and Cognitive Function in Children from Rural Area Staying in Residential School. Indian J. Physiol. Pharmacol..

[34] Nemati A., Barak M., Dehgan M.H., Alimohammadi H., Ettehad G.H., Baghi N., Arvin J., Mogadam R.A., Abbasgholizade N. (2007). Relation between Iron Deficiency and Anemia Whit School Success, Weight and Height in Schoolgirls Aged 12 Year Old in Ardebil Province of Iran, 2005. Res. J. Biol. Sci..

[35] Olson C.L., Acosta L.P., Hochberg N.S., Olveda R.M., Jiz M., McGarvey S.T., Kurtis J.D., Bellinger D.C., Friedman J.F. (2009). Anemia of Inflammation Is Related to Cognitive Impairment among Children in Leyte, The Philippines. PLoS Negl. Trop. Dis..

[36] Ortega R.M., Gonzalez Fernandez M., Paz L., Andres P., Jimenez L.M., Jimenez M.J., Gonzalez Gross M., Requejo A.M., Gaspar M.J. (1993). Influence of Iron Status on Attention and Intellectual Performance of a Population of Spanish Adolescents. Arch. Lat. Nutr..

[37] Sen A., Kanani S.J. (2006). Deleterious Functional Impact of Anemia on Young Adolescent School Girls. Indian Pediatr..

[38] SoonMyung H., HyeJin H., HyunHee K. (2004). A Study on Iron Nutritional Status and Anemia of Middle School Girls in Ulsan Metropolitan City. J. Community Nutr..

[39] Teni M., Shiferaw S., Asefa F. (2017). Anemia and Its Relationship with Academic Performance among Adolescent School Girls in Kebena District, Southwest Ethiopia. Biotechnol. Health Sci..

[40] Thalanjeri P., Karanth H., Vinutha Shankar M.S., Kutty K. (2016). Impact of Iron Deficiency Anemia on Cognition of School Children of South India. Indian J. Clin. Anat. Physiol..

[41] Walker S.P., Grantham-Mcgregor S.M., Himes J.H., Williams S., Duff E.M. (1998). School Performance in Adolescent Jamaican Girls: Associations with Health, Social and Behavioural Characteristics, and Risk Factors for Dropout. J. Adolesc..

[42] Webb T.E., Oski F.A. (1973). Iron Deficiency Anemia and Scholastic Achievement in Young Adolescents. J. Pediatr..

[43] Kalaichelvi D. (2016). A Study on Effectiveness of Nutritional Intervention in Treating Iron Deficiency Anemia and Improving Intelligence among Adolescent Girls. Ph.D. Thesis.

[44] Khan M.A., Farhana Haseen F., Jalal C.S.B., Rahman M., Akter S., Huda S.N. Effects of a Multiple Micronutrient Beverage Supplement on Haematologic, Iron, Vitamin A and Growth Status and Cognitive Development and School Performance among Adolescent Girls in Bangladesh. BRAC; 2004. https://idl-bnc-idrc.dspacedirect.org/handle/10625/32143.

[45] Muthayya S., Thankachan P., Hirve S., Amalrajan V., Thomas T., Lubree H., Agarwal D., Srinivasan K., Hurrell R.F., Yajnik C.S. (2012). Iron Fortification of Whole Wheat Flour Reduces Iron Deficiency and Iron Deficiency Anemia and Increases Body Iron Stores in Indian School-Aged Children. J. Nutr..

[46] Scott S.P., Murray-Kolb L.E., Wenger M.J., Udipi S.A., Ghugre P.S., Boy E., Haas J.D. (2018). Cognitive Performance in Indian School-Going Adolescents Is Positively Affected by Consumption of Iron-Biofortified Pearl Millet: A 6-Month Randomized Controlled Efficacy Trial. J. Nutr..

[47] Solon F.S., Sarol J.N., Bernardo A.B.I., Solon J.A.A., Mehansho H., Sanchez-Fermin L.E., Wambangco L.S., Juhlin K.D. (2003). Effect of a Multiple-Micronutrient-Fortified Fruit Powder Beverage on the Nutrition Status, Physical Fitness, and Cognitive Performance of Schoolchildren in the Philippines. Food Nutr. Bull..

[48] Vinodkumar M., Erhardt J.G., Rajagopalan S. (2009). Impact of a Multiple-Micronutrient Fortified Salt on the Nutritional Status and Memory of Schoolchildren. Int. J. Vitam. Nutr. Res..

[49] Ballin A., Berar M., Rubinstein U., Kleter Y., Hershkovitz A., Meytes D. (1992). Iron State in Female Adolescents. Am. J. Dis. Child..

[50] Bruner A.B., Joffe A., Duggan A.K., Casella J.F., Brandt J. (1996). Randomised Study of Cognitive Effects of Iron Supplementation in Non-Anaemic Iron-Deficient Adolescent Girls. Lancet.

[51] Chellappa A.R., Karunanidhi S. (2012). Effect of Iron and Zinc Supplementation on Cognitive Functions of Female Adolescents in Chennai, India. Int. Proc. Chem. Biol. Environ. Eng. (IPCBEE).

[52] Lambert A., Knaggs K., Scragg R., Schaaf D. (2002). Effects of Iron Treatment on Cognitive Performance and Working Memory in Non-Anaemic, Iron-Deficient Girls. N. Z. J. Psychol..

[53] Rezaeian A., Ghayour-Mobarhan M., Mazloum S.R., Yavari M., Jafari S.A. (2014). Effects of Iron Supplementation Twice a Week on Attention Score and Haematologic Measures in Female High School Students. Singap. Med. J..

[54] Soemantri A.G., Gopaldas T., Seshadri S., Pollitt E. (1989). Preliminary Findings on Iron Supplementation and Learning Achievement of Rural Indonesian Children. Am. J. Clin. Nutr..

[55] Soemantri A.G., Pollitt E., Kim I. (1985). Iron Deficiency Anemia and Educational Achievement. Am. J. Clin. Nutr..

[56] Umamaheswari K., Bhaskaran M., Krishnamurthy G., Kavita V. (2011). Effect of Iron and Zinc Deficiency on Short Term Memory in Children. Indian Pediatr..

[57] Haskell C.F., Scholey A.B., Jackson P.A., Elliott J.M., Defeyter M.A., Greer J., Robertson B.C., Buchanan T., Tiplady B., Kennedy D.O. (2008). Cognitive and Mood Effects in Healthy Children during 12 Weeks’ Supplementation with Multi-Vitamin/Minerals. Br. J. Nutr..

[58] Lynn R., Harland E.P. (1998). A Positive Effect of Iron Supplementation on the IQs of Iron Deficient Children. Personal. Individ. Differ..

[59] Nelson M., Naismith D.J., Burley V., Gatenby S., Geddes N. (1990). Nutrient Intakes, Vitamin-Mineral Supplementation, and Intelligence in British Schoolchildren. Br. J. Nutr..

[60] Schoenthaler S.J., Amos S.P., Eysenck H.J., Peritz E., Yudkin J. (1991). Controlled Trial of Vitamin-Mineral Supplementation: Effects of Intelligence and Performance. Personal. Individ. Differ..

[61] Southon S., Wright A.J.A., Finglas P.M., Bailey A.L., Loughridge J.M., Walker A.D. (1994). Dietary Intake and Micronutrient Status of Adolescents: Effect of Vitamin and Trace Element Supplementation on Indices of Status and Performance in Tests of Verbal and Non-Verbal Intelligence. Br. J. Nutr..

[62] Vinodkumar M., Rajagopalan S., Vinodkumar M., Rajagopalan S. (2009). Efficacy of Fortification of School Meals with Ferrous Glycine Phosphate and Riboflavin against Anemia and Angular Stomatitis in Schoolchildren. Food Nutr. Bull..

[63] The World Bank World Bank Country and Lending Groups. https://datahelpdesk.worldbank.org/knowledgebase/articles/906519-world-bank-country-and-lending-groups.

[64] Deary I.J., Pattie A., Starr J.M. (2013). The Stability of Intelligence from Age 11 to Age 90 Years: The Lothian Birth Cohort of 1921. Psychol. Sci..

[65] Plomin R., von Stumm S. (2018). The New Genetics of Intelligence. Nature reviews. Genetics.

[66] Walker S., Grantham-McGregor S., Powell C., Chang S. (2000). Effects of Growth Restriction in Early Childhood on Growth, IQ, and Cognition at Age 11 to 12 Years and the Benefits of Nutritional Supplementation and Psychosocial Stimulation. J. Pediatr..

[67] Khor G.L., Misra S. (2012). Micronutrient Interventions on Cognitive Performance of Children Aged 5-15 Years in Developing Countries. Asia Pac. J. Clin. Nutr..

[68] Lam L.F., Lawlis T.R. (2017). Feeding the Brain—The Effects of Micronutrient Interventions on Cognitive Performance among School-Aged Children: A Systematic Review of Randomized Controlled Trials. Clin. Nutr..

[69] Finkelstein J.L., Fothergill A., Hackl L.S., Haas J.D., Mehta S. (2019). Iron Biofortification Interventions to Improve Iron Status and Functional Outcomes. Proc. Nutr. Soc..

[70] Grantham-McGregor S., Ani C. (2001). A Review of Studies on the Effect of Iron Deficiency on Cognitive Development in Children. J. Nutr..

[71] Pasricha S., Gheorghe A., Sakr-Ashour F., Arcot A., Neufeld L., Murray-Kolb L., Suchdev P., Bode M. (2020). Net Benefit and Cost-Effectiveness of Universal Iron-Containing Multiple Micronutrient Powders for Young Children in 78 Countries: A Microsimulation Study. Lancet Glob. Health.

[72] UNESCO Institute of Statistics Data for the Sustainable Development Goals. http://uis.unesco.org/.

[73] UNESCO Institute of Statistics Learning Outcomes. http://uis.unesco.org/en/topic/learning-outcomes.

[74] Nutrition International Helping Adolescent Girls in Indonesia Reach Their Full Potential. https://www.nutritionintl.org/project/mitra-youth/.

[75] United Nations (2021). Children’s Fund (UNICEF) Programming Guidance: Nutrition in Middle Childhood and Adolescence.

[76] Bhardwaj A., Murage L., Sharma S., Dipo D., Makena C., Roche M., Arabi M. (2021). Weekly Iron and Folic Acid Supplementation and Nutrition Education for Adolescent Girls in Africa and Asia. Field Exch..

[77] Roche M.L., Bury L., Yusadiredja I.N., Asri E.K., Purwanti T.S., Kusyuniati S., Bhardwaj A., Izwardy D. (2018). Adolescent Girls’ Nutrition and Prevention of Anaemia: A School Based Multisectoral Collaboration in Indonesia. BMJ.

[78] McCulloch A., Joshi H. (2001). Neighbourhood and Family Influences on the Cognitive Ability of Children in the British National Child Development Study. Soc. Sci. Med..

[79] Edefonti V., Rosato V., Parpinel M., Nebbia G., Fiorica L., Fossali E., Ferraroni M., Decarli A., Agostoni C. (2014). The Effect of Breakfast Composition and Energy Contribution on Cognitive and Academic Performance: A Systematic Review. Am. J. Clin. Nutr..

[80] Wachs T.D. (1999). The Nature and Nurture of Child Development. Food Nutr Bull..

[81] World Health Organization (2016). Guideline: Daily Iron Supplementation in Adult Women and Adolescent Girls.

[82] World Health Organization (2016). Guideline: Daily Iron Supplementation in Infants and Children.

[83] World Health Organization (2011). Guideline: Intermittent Iron and Folic Acid Supplementation in Menstruating Women.

[84] Samson K.L.I., Loh S.P., Lee S.S., Sulistyoningrum D.C., Khor G.L., Mohd Shariff Z., Ismai I.Z., Yelland L.N., Leemaqz S., Makrides M. (2020). Weekly Iron-Folic Acid Supplements Containing 2.8 Mg Folic Acid Are Associated with a Lower Risk of Neural Tube Defects than the Current Practice of 0.4 Mg: A Randomised Controlled Trial in Malaysia. BMJ Glob. Health.

[85] Roche M.L., Samson K.L.I., Karakochuk C.D., Green T.J., Martínez H. (2021). Perspective: Weekly Iron and Folic Acid Supplementation (WIFAS): A Critical Review and Rationale for Inclusion in the Essential Medicines List to Accelerate Anemia and Neural Tube Defects Reduction. Adv. Nutr..

[86] Nutrition International Women and Girls’ Nutrition. https://www.nutritionintl.org/what-we-do/by-programs/women-girls-nutrition/.

[87] De-Regil L.M., Jefferds M.E.D., Sylvetsky A.C., Dowswell T. (2011). Intermittent Iron Supplementation for Improving Nutrition and Development in Children under 12 Years of Age. Cochrane Database Syst. Rev..

[88] Low M.S.Y., Speedy J., Styles C.E., De-Regil L.M., Pasricha S.-R. (2016). Daily Iron Supplementation for Improving Anaemia, Iron Status and Health in Menstruating Women. Cochrane Database Syst. Rev..

[89] Fernandez-Gaxiola A., De-Regil L. (2019). Intermittent Iron Supplementation for Reducing Anaemia and Its Associated Impairments in Adolescent and Adult Menstruating Women. Cochrane Database Syst. Rev..

[90] De-Regil L.M., Jefferds M.E.D., Peña-Rosas J.P. (2017). Point-of-Use Fortification of Foods with Micronutrient Powders Containing Iron in Children of Preschool and School-Age. Cochrane Database Syst. Rev..

[91] Das J.K., Salam R.A., Mahmood S.B., Moin A., Kumar R., Mukhtar K., Lassi Z.S., Bhutta Z.A. (2019). Food Fortification with Multiple Micronutrients: Impact on Health Outcomes in General Population. Cochrane Database Syst. Rev..

[92] de Jager C.A., Dye L., de Bruin E.A., Butler L., Fletcher J., Lamport D.J., Latulippe M.E., Spencer J.P., Wesnes K. (2014). Criteria for Validation and Selection of Cognitive Tests for Investigating the Effects of Foods and Nutrients. Nutr. Rev..

[93] World Health Organization (2011). Haemoglobin Concentrations for the Diagnosis of Anaemia and Assessment of Severity.

