# Supplementary material for: Iron Status, Anemia, and Iron Interventions and Their Associations with Cognitive and Academic Performance in Adolescents: A Systematic Review
Source: Nutrients. 2022 Jan 5;14(1):224. doi: 10.3390/nu14010224 (PMC8746955; doi:10.3390/nu14010224)
Supplement: Supplementary file 1 [file nutrients-14-00224-s001.zip › nutrients-1498904-supplementary.pdf]

ONLINE SUPPLEMENTARY MATERIAL

Table of Contents

Supplemental Table S1. Medline search strategy for 1946 to December 05, 2019

Supplemental Table S2. EMBASE search strategy for 1974 to December 05, 2019

Supplemental Table S3. CINAHL search strategy for 1982 to December 05, 2019

Supplemental Table S4. PRISMA checklist

Table S1. Medline search strategy for 1946 to December 05, 2019

Search No.	Search	Results	Search Type
1	Adolescent/ or Child/	2740059	Advanced
2	(adolescen* or teen* or "young adult*" or "young people*" or "school child*" or "school-aged" or schoolchild* or "middle school*" or pubescen* or juvenile* or youth* or "high school*" or pre-pubesc* or prepubesc*).ti,ab.	537210	Advanced
3	1 or 2	2941240	Advanced
4	exp cognition/ or executive function/ or exp learning/ or Social Behavior/ or Social Learning/ or exp thinking/ or attention/	752960	Advanced
5	(cogniti* or "executive function*" or learn* or "social behavio?r" or think* or attention* or "social develop*").ti,ab.	1107348	Advanced
6	exp Educational Measurement/	144022	Advanced
7	((education* or academic*) adj2 ("test score*" or success* or achiev* or measure* or perform*)).ti,ab.	18090	Advanced
8	4 or 5 or 6 or 7	1692449	Advanced
9	Anemia, Iron-Deficiency/ or Iron/ or Iron, Dietary/	99266	Advanced
10	(iron or an?emia).ti,ab.	292102	Advanced
11	9 or 10	317548	Advanced
12	3 and 8 and 11	1561	Advanced
13	limit 12 to humans	1425	Advanced

Table S2. EMBASE search strategy for 1974 to December 05, 2019

Search No.	Search	Results	Search Type
1	Adolescent/ or Child/	2463816	Advanced
2	(adolescen* or teen* or "young adult*" or "young people*" or "school child*" or "school-aged" or schoolchild* or "middle school*" or pubescen* or juvenile* or youth* or "high school*" or pre-pubesc* or prepubesc*).ti,ab.	683225	Advanced
3	1 or 2	2722629	Advanced
4	exp cognition/ or exp cognition assessment/ or exp learning/ or social behavior/ or social learning/ or exp attention/ or exp thinking/	2220849	Advanced
5	cogniti* or "executive function*" or learn* or "social behavio?r" or think* or attention* or "social develop*").ti,ab.	1467827	Advanced
6	exp education/	1377183	Advanced
7	((education* or academic*) adj2 ("test score*" or success* or achiev* or measure* or perform*)).ti,ab.	22538	Advanced
8	4 or 5 or 6 or 7	4008178	Advanced
9	iron deficiency/ or iron depletion/ or iron intake/ or iron deficiency anemia/ or iron therapy/ or iron blood level/ or iron/	174691	Advanced
10	(iron or an?emia).ti,ab.	366708	Advanced
11	9 or 10	421826	Advanced
12	3 and 8 and 11	6600	Advanced
13	limit 12 to humans	6169	Advanced

Table S3. CINAHL search strategy for 1982 to December 05, 2019

Search No.	Search	Results
1	(MH "Adolescence+") OR (MH "Child+")	875317
2	TI adolescen* OR AB adolescen* OR TI teen* OR AB teen* OR TI "young adult*" OR AB "young adult*" OR TI "young people*" OR AB "young people*" OR TI "school child*" OR AB "school child" OR TI "school-aged" OR AB "school-aged" OR TI schoolchild* OR AB schoolchild* OR TI "middle school*" OR AB "middle school*" OR TI pubescen* OR AB pubescen* OR TI juvenile* OR AB juvenile* OR TI youth* OR AB youth* OR TI "high school*" OR AB "high school*" OR TI pre-pubesc* OR AB pre-pubesc* OR TI prepubesc* OR AB prepubesc*	215128
3	1 or 2	933635
4	(MH "Cognition+") OR (MH "Executive Function") OR (MH "Learning+") OR (MH "Thinking+") OR (MH "Social Behavior") OR (MH"Attention")	297331
5	TI cogniti* OR AB cogniti* OR TI "executive function*" OR AB "executive function*" OR TI learn* OR AB learn* OR TI think* OR AB think* OR TI attention OR AB attention OR TI "social develop*" OR AB "social develop*" OR TI "social behavio#r" OR AB "social behavio#r"	359458
6	(MH "Outcomes of Education") OR (MH "Education+") OR (MH "Educational Measurement+") OR (MH "Student Performance Appraisal+")	834395
7	(((education* or academic*) n2 ("test score*" or success* or achiev* or measure* or perform*)).ti,ab.	0
8	4 or 5 or 6 or 7	1239762
9	(MH "Iron") OR (MH "Anemia, Iron Deficiency")	9592
10	TI iron OR AB iron OR TI an#emia OR AB an#emia	29256
11	9 or 10	31495
12	3 and 8 and 11	1141
13	limit 12 to humans	754

Table S4. PRISMA Checklist

Section/topic	#	Checklist item	Reported on page #
**TITLE**			
Title	1	Identify the report as a systematic review, meta-analysis, or both.	1
**ABSTRACT**			
Structured summary	2	Provide a structured summary including, as applicable: background; objectives; data sources; study eligibility criteria, participants, and interventions; study appraisal and synthesis methods; results; limitations; conclusions and implications of key findings; systematic review registration number.	2
**INTRODUCTION**			
Rationale	3	Describe the rationale for the review in the context of what is already known.	3-4
Objectives	4	Provide an explicit statement of questions being addressed with reference to participants, interventions, comparisons, outcomes, and study design (PICOS).	4-5
**METHODS**			
Protocol and registration	5	Indicate if a review protocol exists, if and where it can be accessed (e.g., Web address), and, if available, provide registration information including registration number.	4
Eligibility criteria	6	Specify study characteristics (e.g., PICOS, length of follow-up) and report characteristics (e.g., years considered, language, publication status) used as criteria for eligibility, giving rationale.	5
Information sources	7	Describe all information sources (e.g., databases with dates of coverage, contact with study authors to identify additional studies) in the search and date last searched.	4<br>Supplementary file
Search	8	Present full electronic search strategy for at least one database, including any limits used, such that it could be repeated.	Supplementary tables 1-3
Study selection	9	State the process for selecting studies (i.e., screening, eligibility, included in systematic review, and, if applicable, included in the meta-analysis).	5
Data collection process	10	Describe method of data extraction from reports (e.g., piloted forms, independently, in duplicate) and	6

		any processes for obtaining and confirming data from investigators.	
Data items	11	List and define all variables for which data were sought (e.g., PICOS, funding sources) and any assumptions and simplifications made.	6
Risk of bias in individual studies	12	Describe methods used for assessing risk of bias of individual studies (including specification of whether this was done at the study or outcome level), and how this information is to be used in any data synthesis.	5-6
Summary measures	13	State the principal summary measures (e.g., risk ratio, difference in means).	NA
Synthesis of results	14	Describe the methods of handling data and combining results of studies, if done, including measures of consistency (e.g., I^2) for each meta-analysis.	NA
Risk of bias across studies	15	Specify any assessment of risk of bias that may affect the cumulative evidence (e.g., publication bias, selective reporting within studies).	NA
Additional analyses	16	Describe methods of additional analyses (e.g., sensitivity or subgroup analyses, meta-regression), if done, indicating which were pre-specified.	NA
**RESULTS**			
Study selection	17	Give numbers of studies screened, assessed for eligibility, and included in the review, with reasons for exclusions at each stage, ideally with a flow diagram.	6<br>Fig. 1
Study characteristics	18	For each study, present characteristics for which data were extracted (e.g., study size, PICOS, follow-up period) and provide the citations.	7-8<br>Tables 1 & 2
Risk of bias within studies	19	Present data on risk of bias of each study and, if available, any outcome level assessment (see item 12).	8-9
Results of individual studies	20	For all outcomes considered (benefits or harms), present, for each study: (a) simple summary data for each intervention group (b) effect estimates and confidence intervals, ideally with a forest plot.	9-22<br>Tables 1 & 2
Synthesis of results	21	Present results of each meta-analysis done, including confidence intervals and measures of consistency.	NA
Risk of bias across studies	22	Present results of any assessment of risk of bias across studies (see Item 15).	NA

Additional analysis	23	Give results of additional analyses, if done (e.g., sensitivity or subgroup analyses, meta-regression [see Item 16]).	NA
**DISCUSSION**			
Summary of evidence	24	Summarize the main findings including the strength of evidence for each main outcome; consider their relevance to key groups (e.g., healthcare providers, users, and policy makers).	22-26
Limitations	25	Discuss limitations at study and outcome level (e.g., risk of bias), and at review-level (e.g., incomplete retrieval of identified research, reporting bias).	26-27
Conclusions	26	Provide a general interpretation of the results in the context of other evidence, and implications for future research.	27-29
**FUNDING**			
Funding	27	Describe sources of funding for the systematic review and other support (e.g., supply of data); role of funders for the systematic review.	1
